# Active cell divisions generate fourfold orientationally ordered phase in living tissue

**DOI:** 10.1038/s41567-023-02025-3

**Published:** 2023-05-01

**Authors:** Dillon J. Cislo, Fengshuo Yang, Haodong Qin, Anastasios Pavlopoulos, Mark J. Bowick, Sebastian J. Streichan

**Affiliations:** 1 Department of Physics, University of California, Santa Barbara, Santa Barbara, CA, USA; 2 Center for Studies in Physics and Biology, Rockefeller University, New York, NY, USA; 3 The T.C. Jenkins Department of Biophysics, Johns Hopkins University, Baltimore, MD, USA; 4 Department of Physics, University of California, San Diego, La Jolla, CA, USA; 5 Institute of Molecular Biology and Biotechnology, Foundation for Research and Technology Hellas, Heraklion, Greece; 6 Kavli Institute for Theoretical Physics, University of California, Santa Barbara, Santa Barbara, CA, USA

## Abstract

Morphogenesis, the process through which genes generate form, establishes tissue-scale order as a template for constructing the complex shapes of the body plan. The extensive growth required to build these ordered substrates is fuelled by cell proliferation, which, naively, should destroy order. Understanding how active morphogenetic mechanisms couple cellular and mechanical processes to generate order–rather than annihilate it–remains an outstanding question in animal development. We show that cell divisions are the primary drivers of tissue flow, leading to a fourfold orientationally ordered phase. Waves of anisotropic cell proliferation propagate across the embryo with precise patterning. Defects introduced into the nascent lattice by cell divisions are moved out of the tissue bulk towards the boundary by subsequent divisions. Specific cell proliferation rates and orientations enable cell divisions to organize rather than fluidize the tissue. We observe this using live imaging and tissue cartography to analyse the dynamics of fourfold tissue ordering in the trunk segmental ectoderm of the crustacean *Parhyale hawaiensis* beginning 72 h after egg lay. The result is a robust, active mechanism for generating global orientational order in a non-equilibrium system that sets the stage for the subsequent development of shape and form.

Ordered cellular geometries in developing tissues serve as patterned substrates from which complex arrangements of body parts can be built. The crucial organizing role that order plays in morphogenesis is particularly apparent in direct developers. These animals assemble a complete, miniature version of the adult body during embryogenesis^1^. The limbs and organs comprising the adult form are arranged according to specific body plans that ensure proper biomechanical functionality^2,3^. Body parts develop with ordered placements and are aligned and oriented relative to distinct principal body axes^3^. To reliably generate the correct arrangements of limbs and organs, direct developers create organizational templates from ordered regions of tissue, akin to a coordinate system spanning the entire body.

Such templates must be ordered to delineate the body plan, but also retain sufficient fluidity to facilitate the large deformations necessary during development. Orientational order, an intermediate state between solid and liquid matter, has been previously studied in non-living, thermally equilibrated systems^4–8^. More recently, orientational order has been demonstrated in the late stages of development, where organs use planar polarized signals to arrange cells into an ordered phase in the absence of proliferation^9–12^. In contrast, the initial structuring of the body plan during early embryonic stages occurs via the sequential outgrowth of segments and is fuelled by cell proliferation^1^. Generically, cell proliferation should give rise to fluid-like rearrangements that mix cells and prevent the initially fluid tissue from ever achieving an ordered stat^13–15^. Orientationally ordered phases occupy only small fractions of their respective phase spaces in thermally equilibrated systems^16–20^. Thus, it remains unclear how non-equilibrium mechanisms in living systems can generate the requisite order to specify the body plan in the presence of cell divisions.

Here we used *Parhyale hawaiensis*, an emerging model system of direct limb morphogenesis^21,22^, to study the interplay of growth and order. *Parhyale* sequentially implements its body plan via extensive cell proliferation^23^. Before appendage outgrowth, the ectoderm forms a grid of locally ordered cells^23,24^ (Fig. 1a,c–e), a feature shared among malacostracans^22^. The rows of this grid correspond to segments of the adult body^23,24^. Limb buds form at specific locations in the grid and give rise to numerous functionally specific appendages^21^. Importantly, limb orientation, in terms of the dorsal–ventral (D−V) and anterior–posterior (A−P) axes, can be traced back to the local arrangement of precursor cells at the grid stage^23,25^.

We performed the *in toto* live imaging of four *Parhyale* embryos using multiview light-sheet microscopy^26^ over a 35h window beginning three days after egg lay (AEL) (Fig. 1a) (Methods provides more details on data curation). During this period, the ectoderm is a monolayer^23^ and can be well approximated as a curved two-dimensional (2D) surface. Using tissue cartography^27,28^, the surface of interest corresponding to the ectoderm was dynamically extracted at each time point (Methods, Supplementary Fig. 1 and Supplementary Video 1). This curved surface was then conformally mapped into the plane so that the relative cell orientations, and therefore the orientational order, could be faithfully quantified from the planar data (Fig. 1b–e and Supplementary Fig. 2). Three-dimensional (3D) and 2D dynamic visualizations of the growth process are shown in Supplementary Videos 2–4.

Working in the 2D conformal parameterization space, we implemented automated image segmentation routines to detect cells (Methods). We found that the number of cells increases exponentially with a typical doubling time of ~10 h (Fig. 1f). Next, we constructed a complex order parameter to quantify the relative orientations of neighbouring cells (Fig. 2a). The cell positions were taken to be the centres of mass of the nuclei. Instantaneous cell–cell connectivity was approximated by Voronoi tessellation (Supplementary Fig. 3). The order parameter assigns a pair of quantitative measures, magnitude and phase, to each cell indicating the extent to which neighbouring cells are coherently positioned according to a specific n-fold lattice structure and the local orientation of the ordered neighbourhood, respectively (Supplementary Fig. 4). Explicitly, the n-fold order parameter of cell j is given by

(1) $$\psi_{n}(j)=\frac{1}{\sum_{k}\ell_{jk}^{2}}\sum_{k\in 𝒩(j)}\ell_{jk}^{2}{\text{e}}^{\text{i}n\theta_{jk}},$$

where the sum runs over all the nearest neighbours k of cell j, that is, $k\in 𝒩(j);\theta_{jk}$ is the angle formed by the separation vector between cells j and k and the horizontal axis in two dimensions; and $\ell_{jk}$ denotes the length of the Voronoi edge shared by cells j and k. The magnitude of the order parameter ranges between 0 (no n-fold order) and 1 (maximum n-fold order). For n=4, that is, fourfold order parameters, the magnitude of the order parameter peaks when all the neighbours of a cell are organized in a rectangular fashion (Fig. 2b,c). Our analysis reveals that the tissue initially exhibits no fourfold order (Fig. 2d,e). Gradually, as the number of cells grows, the tissue adopts an increasingly fourfold ordered state, which peaks in magnitude at around 82.5h AEL (Fig. 2d,f).

We also calculated the two-point correlation functions of the order parameter, namely, $C_{n}(r)=<\psi_{n}(r)\psi_{n}^{*}(0)>(n=4,6)$, which measure the agreement of the magnitude and phase of local order between cells as a function of their separation (Fig. 2g and Supplementary Figs. 5a and 6a,b). At early times, orientational order is short ranged, restricted to less than a cell length. In contrast, at later times, when the global fourfold order parameter peaks, the orientational order is quasi-long ranged, with correlations that decay algebraically across the entire surface (Fig. 2g and Supplementary Fig. 6a). Therefore, strongly ordered local cell neighbourhoods are coherently ordered–in both magnitude and phase–across the whole embryo. No substantial sixfold order was detected at any time during this stage (Fig. 2d and Supplementary Figs. 5a and 6b). In particular, although the global sixfold order parameter transiently rises to a modest $\left|<\psi_{6}>\right|\approx 0.3$ (for comparison, the sixfold order parameter observed in a hexagonally ordered arrangement of hair cells in the mammalian inner ear peaks at ≳0.7 (ref. 12)), the sixfold order correlations always decay exponentially. Exponentially decaying correlation functions categorically prohibit the possibility of a hexatically ordered phase^16^. Next, we tested if the ectodermal grid exhibits positional order using the pair correlation function g(Δr), that is, the high-resolution histogram of distances between pairs of cells^16,20^. Isotropic correlations g(r) decay exponentially (Supplementary Figs. 5b and 6c) and the cell spacing is not periodic along any single axis (Supplementary Fig. 5c,d). Thus, the tissue exhibits neither positional nor smectic order. More details about the construction of the various correlation functions, including the validation of results against synthetic datasets (Supplementary Figs. 7 and 8 and Supplementary Table 1) and a discussion of finite size effects (Supplementary Fig. 9), can be found in Methods.

This lack of periodic cell spacing can be partly explained by the presence of edge defects in rows of cells. Edge defects are locations where a lattice row freely terminates in the interior, for example, three rows become two. In elasticity theory, such defects are called ‘dislocations’ and are well known to disrupt translational order, but preserve orientational order^29^. In the *Parhyale* germband, these defects are disordered, reminiscent of the defect gases characteristic of orientationally ordered phases (Fig. 2h). Cell divisions are the primary mechanism mediating both defect generation and subsequent defect dynamics within the tissue (Supplementary Video 5). Although defects are generally isolated during the time the tissue peaks in orientational order, many defects can be associated with one another by a row of ordered cells with a defect on the left and right sides, respectively (Fig. 2h). Together, these results show that the tissue achieves a true orientationally ordered phase extending over the entire trunk ectodermal germband.

We performed single-cell tracking to reconstruct the flow fields that organize the ectoderm during the rise of fourfold order. A parasegment precursor row (PSPR) is the fundamental supercellular unit of morphogenesis in the trunk ectodermal germband during this phase of growth^23,24^. A PSPR is a single row of cells, oriented perpendicular to the A−P axis, which can be directly associated with particular segments of the adult body. As a unit, PSPRs are sequentially appended to the grid. Cells are recruited from a pool of unorganized ectoderm at the posterior pole of the embryo and assembled into nascent rows so that each newly constructed PSPR lies immediately posterior to the PSPR that preceded it (Fig. 3a). Despite being arranged as a row and, in general, not containing any edge defects (Supplementary Video 5), newly built PSPRs do not yet exhibit fourfold order (Fig. 2d and Supplementary Fig. 10).

Once a PSPR is assembled, its constituent cells undergo two rounds of highly choreographed cell divisions. Within each parasegment, mitotic waves are initiated at the ventral midline and spread outwards towards the dorsal regions of the embryo (Fig. 3b and Supplementary Videos 6 and 7). The timing of these intrasegment waves is stereotypic among all the segments (Fig. 3f and Supplementary Fig. 11c); since PSPRs are sequentially constructed, their onset is staggered between adjacent PSPRs (Fig. 3d,e and Supplementary Fig. 11a,b). This choreography leads to two orthogonal phase waves of cell divisions with distinct wave velocities: a fast wave within each PSPR spreading ventral to dorsal and a slower one across PSPRs that moves anterior to posterior (Fig. 3c). After completing these two mitotic waves, each PSPR subsequently undergoes rapid differential cleavage in localized regions adjacent to the ventral midline (Fig. 3b). Cells divide faster during differential cleavage than during mitotic waves (Supplementary Fig. 12). Together, these results indicate that PSPRs behave as weakly coupled, independent units running the same modular proliferation program. In other words, cells in different PSPRs begin to divide at different times, but the relative timing of division waves is shared among the segments.

The orientations of divisions comprising the mitotic waves are tightly distributed about the A–P axis (Fig. 3g). This coherence of cell division axes appears to be actively maintained. We frequently found that condensed nuclei with the wrong orientation would rapidly rotate to align with the global division axis (Fig. 3h and Supplementary Video 8). In fact, 7.4% of the 770 tracked division events underwent a reorientation of more than $45^{\circ }$ in the five minutes before division. This patterning of oriented and wave-like timed cell divisions ensures that the defects inserted into the lattice by cell division are effectively ferried out of the tissue towards the boundary (Fig. 3i). Explicitly, as the mitotic waves gradually insert new rows into the bulk of the grid, the incomplete rows manifest defects at their left and right edges (Fig. 2h). These defects are pushed out towards the dorsal regions of the tissue, leaving behind an intact fourfold ordered grid. This type of defect motion is known as ‘defect climb’. It can be contrasted against ‘defect glide’, another type of defect motion, where defects would instead jump between adjacent PSPRs. In non-living systems, glide generally dominates climb since gliding only requires simple updates to the connectivity of nearest neighbours, whereas climb requires the creation of vacancies or interstitial defects^30^. Defect climb in non-living systems typically only becomes the dominant mode of defect motion at extremely high temperatures^31^. Here the presence of cell divisions excites defect climb at room temperature, such that defects, which would otherwise disorder the lattice, are healed by subsequent divisions.

Next, we investigated how the division choreography dynamically shapes the ectoderm at the tissue scale. For the purposes of this analysis, we focused on a subset of six PSPRs. We determined that growth proceeds in two stages (Fig. 4a). In the first stage, mitotic waves extend the germband by inserting new rows without changing the average cell density (Fig. 4b,c and Supplementary Fig. 13). The tissue elongates along the A–P axis and increases in the total area, but its width remains approximately constant. In the second stage, the tissue undergoes convergent extension. Its width is sharply pinched, but its length continues to increase in such a way that the total tissue area is held approximately fixed. Since the rate of cell division remains constant throughout both stages (Fig. 1f), the average cell density necessarily increases during convergent extension.

These two stages also feature markedly different dynamics of the global order parameter (Fig. 2d). During the first stage, when the large-scale motion of the tissue appears to be dominated by mitotic waves, the global order parameter consistently increases. In the second stage, where the tissue experiences a reduction in width that is not explained by the mitotic waves alone, the global order parameter is initially static, but slowly falls off.

Having separately elucidated the kinetics of cell proliferation and the time course of tissue geometry, our next goal was to explicitly connect local cell behaviours with global tissue shape and flow. We developed a hydrodynamic model that directly links coarse-grained tissue flow to bulk contributions from oriented cell divisions via an active source term^32–36^ :

(2) $$v_{1}\nabla^{2}v+v_{2}\nabla (\nabla \cdot v)=-\nabla \cdot \sigma^{a}\equiv -F^{a},$$

where v denotes the tissue velocities; $\sigma^{a}$ are the active stresses; $F^{a}$ are the corresponding active forces; $v_{1}=\tau_{R}\mu$ is the effective shear viscosity; $v_{2}=\tau_{R}(\mu +\lambda )$ is the effective bulk viscosity; $\tau_{R}$ is a timescale corresponding to mechanical relaxation due to growth/tissue remodelling; and μ and λ are the usual Lamé parameters (Methods and Supplementary Text). For simplicity, we assume that cell divisions are the only source of active stresses. During division, the mitotic spindle within cells generates extensile forces akin to a force dipole^14,37^. These division events push on nearby cells, deforming the dividing cell’s neighbours and generating local plastic strain. The viscoelastic relaxation of this instantaneous strain leads to flow in the surrounding tissue (Fig. 4f). In this way, many division events can combine to create large-scale collective motion. We investigated the extent to which the integrated deformations induced by divisions could collectively account for the observed global tissue flow. We first deduced the average plastic deformation induced by a single cell division by rotating 190 cell division events into a common frame (Fig. 4e and Supplementary Figs. 14 and 15). Informed by the features of this average flow field, individual division events were modelled as circular inclusions, that is, finite regions within which the tissue undergoes a permanent plastic strain^38^, with an orientation chosen to align with each measured division axis. We then solved our model using the finite element method with Dirichlet boundary conditions to predict the tissue flow from the observed cell divisions (Supplementary Fig. 16).

We compared the predicted tissue flow with velocity fields quantified from individual cell tracking. Validations were performed against velocity fields extracted from a single embryo. Measured tissue flow fields were mostly harmonic, irrotational and divergence free, except at cell divisions, where the divergence, curl and Laplacian of the flow fields spiked (Fig. 4g–i and Supplementary Fig. 17). Visually, the flow fields predicted from our model appear strikingly similar (Fig. 4j–l). The velocity residual quantitatively confirms that our simple model accurately predicts both direction and magnitude of the observed tissue flow (Fig. 4m–o) (Methods). During the first phase of growth, velocity residuals are typically below 10% (Fig. 4d and Supplementary Fig. 18). This suggests that about 90% of the flow can be accounted for in terms of cell divisions as the dominant bulk contribution. Velocity residuals subsequently increase moderately during convergent extension, reaching a typical level of around 20%. This suggests that divisions are still the main driver, but other mechanisms, currently not accounted for by the model, provide small but measurable adjustments.

Finally, we directly investigated the role of oriented divisions in mediating both cell- and tissue-scale orientational order by simulating division waves using a simple vertex model (Supplementary Text). In line with the observations of actual cells in the tissue (Fig. 5a), simulated cells were assigned a target shape index of $p_{0}=P/\sqrt{A}=4$, where P is the cell perimeter and A is the cell area, that is, the preferred shape index of a perfect square. The tissue was grown by selecting one cell at a time to divide along an axis drawn from a circular von Mises distribution with variable concentration k (Fig. 5f). The results of a typical division wave simulation are shown in Fig. 5b,c. After a single division wave, all the simulations with division orientation concentrations of k≳4 managed to produce global fourfold order parameters of similar magnitude to those observed in real tissue (Fig. 5d) and exhibited algebraically decaying fourfold orientational correlations over the entire simulated domain (Fig. 5e). Simulations with sufficiently random division orientations destroyed all order within the system (Fig. 5d and Supplementary Fig. 19). Interestingly, the simulations also showed that the wave-like spatiotemporal choreography of division timings was not strictly necessary to generate fourfold order. In fact, although the time course of order through the simulations was markedly different, simulations with random division timings produced almost identical fourfold ordering once all the cells had divided (Fig. 5g). This observation reinforces the conclusion that oriented divisions constitute a robust mechanism for fourfold order generation without the need for micromanaged division timings. Our simulations also provided a platform to directly test the extent to which defects introduced into the ectoderm by cell division mediate the loss of order within the tissue (Fig. 5h).

In this work, we combined mathematical modelling with quantitative flow analysis to show that cell divisions are the primary drivers of global tissue flow during *Parhyale* germband extension. We uncovered a global fourfold bond-orientationally ordered phase that emerges via precisely oriented cell divisions. In a scheme that we call defect-driven morphogenesis, cell proliferation introduces defects into the local tissue structure, which are then mobilized by subsequent cell divisions, ultimately giving rise to a highly ordered cell network (Fig. 5i). The choreography of these events is arranged in a timed mitotic wave, spreading at distinct wave velocities across the A−P and D−V axes. The anisotropic timing of this wave of cell divisions results in defect climb at room temperature. Many defects migrate out of the ectodermal bulk towards the boundary. The sparse set of defects that remain destroy the translational order, but leave the orientational order intact. Defect-driven morphogenesis is an efficient as well as a highly robust mechanism for establishing global orientational order in the presence of cell divisions. Similar to non-living matter^39^, the insertion of new particles is an efficient strategy for exploring regions of configuration space corresponding to orientational order. Provided a preferred axis, order can then be produced by having cells divide according to independent, internal timing mechanisms. This timing does not have to be precise (Supplementary Fig. 12). Order within a local region is preserved as long as most of the cells within that region divide once before any particular cell divides twice.

We showed that defect-driven morphogenesis at the single-cell level relies on tightly oriented cell divisions. Our analysis demonstrated that neither the orientation (Supplementary Fig. 20) nor the timing of cell divisions (Supplementary Figs. 21–23) exhibit strong correlations with mechanical or geometric signals. This suggests that the timing and orientations of cell division are actively instructed by biochemical signals, such as morphogen gradients or planar cell polarity, rather than by mechanical feedback. It would be interesting to investigate the precise nature of these biochemical signals. In addition to whatever biochemical symmetry breaking that may set the division timings and orientations, it would also be interesting to clarify the role of myosin in the system, since heterogeneous distributions of junctional myosin have been shown to be capable of generating similar cobblestone patterns in the absence of cell proliferation^11^.

The challenges associated with the live imaging of *Parhyale* limited the number of datasets that could be included in the quantitative analysis. The robustness of our conclusions, however, is supported by fact that *Parhyale* is a lineage-invariant direct developer, an observation previously inferred from fixed samples^23^ and also confirmed by our cell-tracking analysis. Additionally, conclusions about the behaviour of individual parasegments are drawn from observations with greater reproducibility than the number of datasets alone, since each of the many parasegments was shown to operate as an independent supercellular unit.

For small direct developers, like *Parhyale*, arranging cells in an ordered grid might be one of the very few possibilities to establish a coordinate system in the presence of growth^22^. It is intriguing to speculate whether large embryos, with abundant cell numbers, utilize a similar ordering strategy at the mesoscale when arranging bigger periodic units comprising many cells, such as somites in vertebrates^40^. Future work will investigate how defect-driven morphogenesis differs in implementation between relatively small embryos and embryos with large cell numbers and the relationship between tissue-scale order and shape change (Supplementary Fig. 24).

## Online content

Any methods, additional references, Nature Portfolio reporting summaries, source data, extended data, supplementary information, acknowledgements, peer review information; details of author contributions and competing interests; and statements of data and code availability are available at https://doi.org/10.1038/s41567-023-02025-3.

## Methods

### Light-sheet microscopy

For the live imaging of transgenic *Parhyale* embryos, we utilized a custom-built multiview selective-plane illumination microscope^26^. This microscope has two excitation and two detection branches. Both used water-dipping objectives (App LWD 5× (numerical aperture, 1.1), Nikon Instruments, for detection and CFI Plan Fluor 10× (numerical aperture, 0.3) for excitation). Furthermore, each detection branch consisted of a filter wheel (HS-1032, Finger Lakes Instrumentation), with emission filters (BLP02-561R25, Semrock), tube lens (200mm, Nikon Instruments) and a camera (scientific complementary metaloxide–semiconductor; Hamamatsu Flash 4.0V2 ), with an effective pixel size of 0.262mm. The illumination branches featured a tube lens (200 mm, Nikon Instruments), scan lens (S4LFT0061/065, Sill Optics), galvanometric mirror (6,215h, Cambridge Technology) and discrete laser line (561LS OBIS, 561nm). The optical section employed a translation stage from Physik Instrumente (P-629.1CD with E-753 controller), rotation stage (U-628.03 with C-867 controller) and linear actuator (M-231.17 with C-863 controller).

### Data post-processing and microscope automation

To operate the microscope, we used μManager^41^, installed on a Supermicro 7047GR-TF server, with a 12-core Intel Xeon 2.5GHz,64 GB PC3 RAM and hardware RAID level 0 with seven 2.0 TB SATA hard drives. For each sample, we recorded four views, separated by $90^{\circ }$-rotated views, with optical sectioning of 2μm and temporal resolution of 5min. We embedded the embryos in agarose-containing beads as a diagnostic specimen. This was used to register individual views into a common frame by utilizing the Fiji multiview deconvolution plugin^42^, resulting in a final image with an isotropic resolution of 0.2619μm.

### Dataset curation for quantitative analysis

**Fig. 2 |:**
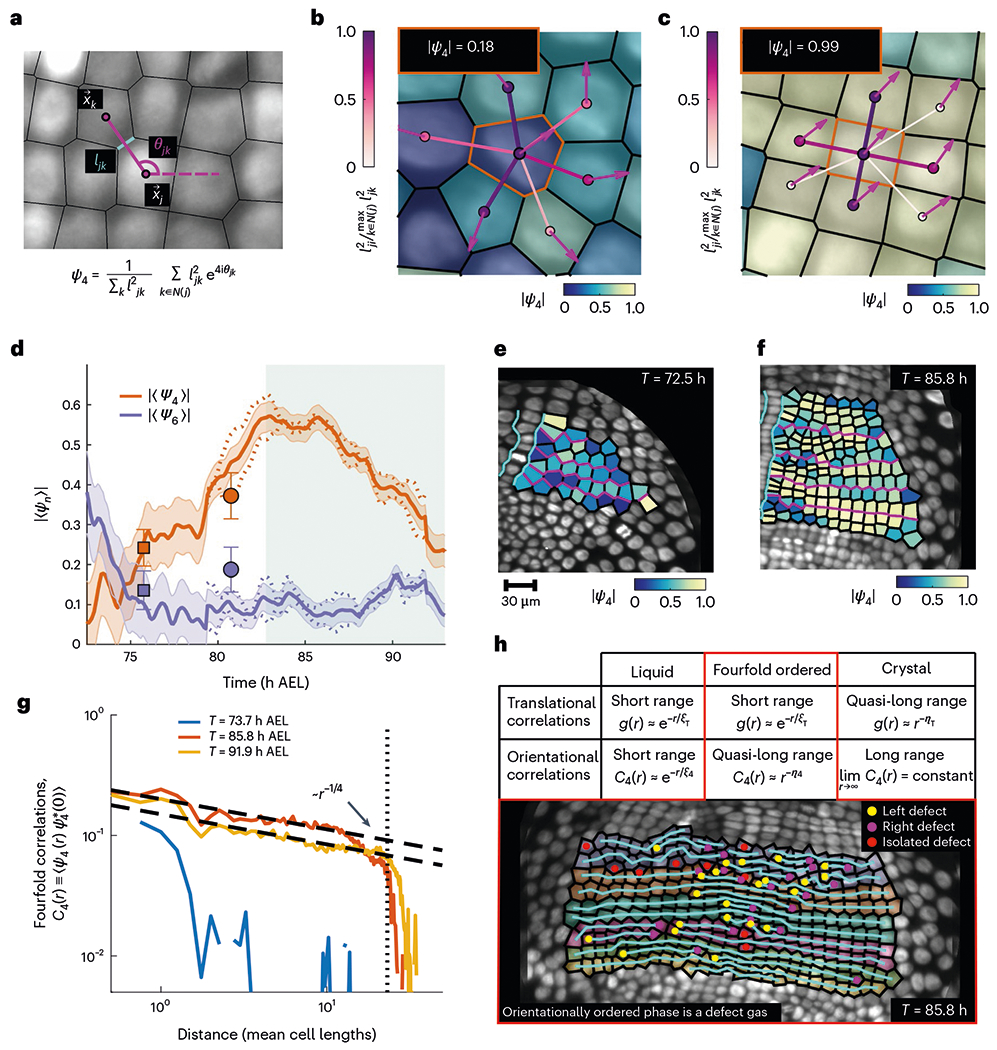
Dynamics of a fourfold orientationally ordered phase in living tissue. **a**, Schematic of the discrete fourfold complex orientational order parameter $\psi_{4}$. Here ${\vec{x}}_{j}$ and ${\vec{x}}_{k}$ denote the centroids of cells j and k, respectively; $\theta_{jk}$ denotes the angle between the horizontal axis and separation vector ${\vec{x}}_{k}-{\vec{x}}_{j}$; and $\ell_{jk}$ denotes the length of the Voronoi edge shared by cells j and k. The sum runs over all the cells *k* in the neighbourhood of cell *j*, that is, k∈𝒩(j). **b**,**c**, Specific examples of the construction of a single-cell order parameter. The value shown in the inset is the magnitude of the order parameter of the seed cell highlighted by an orange boundary. The cell colour and arrows indicate the magnitude and phase of the single-cell order parameters, respectively (colour bar at the bottom right). The colour and thickness of the bonds between the seed cell and its neighbours indicate the relative weight with which each bond contributes to the sum defining the order parameter (colour bar at the left): a disordered cell (**b**); a highly ordered cell (**c**). **d**, Absolute value of the mean fourfold and sixfold orientational order parameters over the whole embryo. The shaded contours show the standard errors. Data are aggregated from four embryos. The dotted lines indicate the measured values from nuclear movies, whereas the markers indicate values measured from the snapshots of membrane marker movies (Methods). The error bars on the snapshot markers denote standard errors. The shaded region highlights the convergent extension phase of growth (Fig. 4). **e**,**f**, Absolute value of a single-cell fourfold orientational order parameter in the left ectodermal compartment at 72.5 h AEL (**e**) and 85.8 h AEL (**f**). The cyan boundaries demarcate the ventral midline cells and the magenta lines delineate the parasegment identities. **g**, Two-point correlator of the fourfold orientational order parameter $C_{4}(r)\equiv <\psi_{4}(r)\psi_{4}^{*}(0)>$. The vertical line shows the largest lateral dimension of the system. The measured values are extracted from a single embryo. The orientational correlations for a second embryo are shown in Supplementary Fig. 6. Both black dashed lines display algebraic decay $r^{-1/4}$ and are intended to be a guide for the eye. **h**, Table enumerating the types of translational and orientational correlations associated with the different material phases. The translational correlations are measured using the pair correlation function g(r). Note that unlike a crystal, an orientationally ordered phase is characterized by a gas of defects. The image in the inset shows the edge defects introduced into rows of cells by division during a time when the germband is fourfold ordered. The cyan lines are a visual guide to distinguish the rows of cells and terminate either on the region boundary or at a left/right defect.

**Fig. 4 |:**
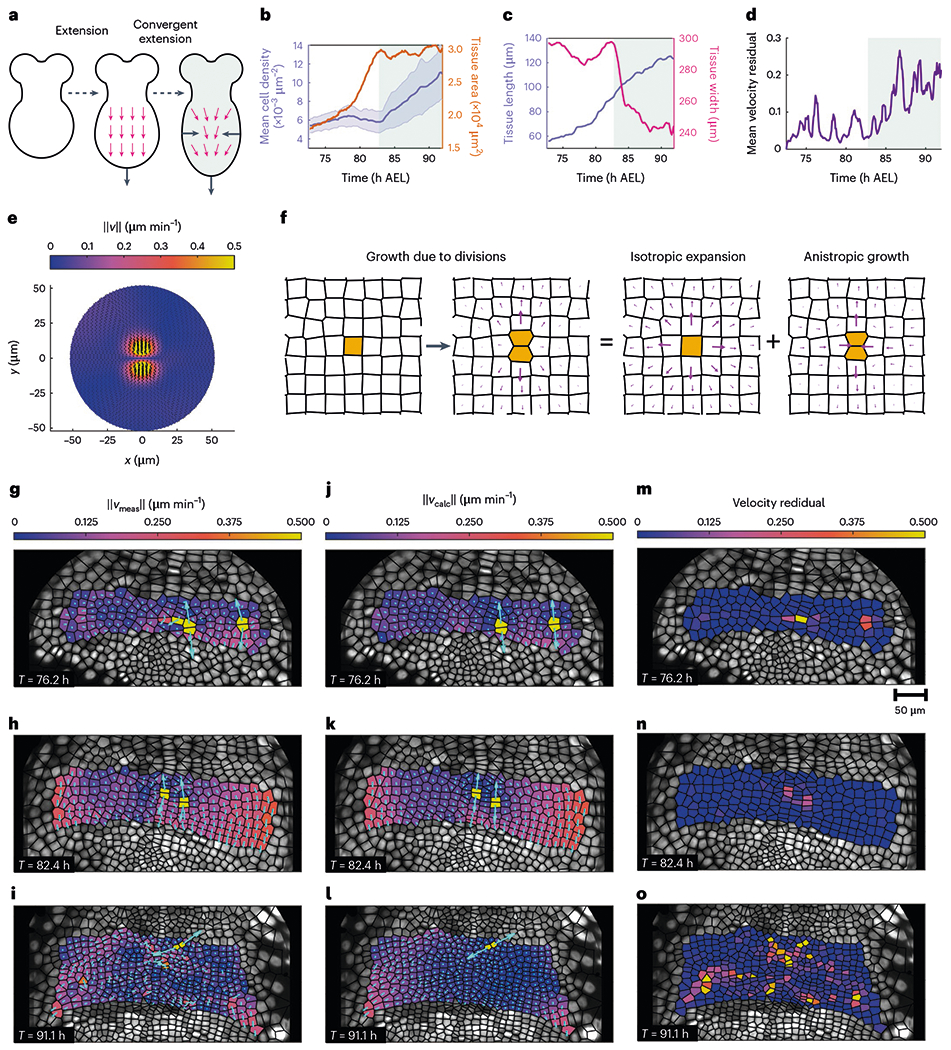
Active cell divisions dictate tissue velocities during germband extension. **a**, Schematic of the two stages of tissue-scale growth observed in the germband. The dashed black arrows indicate the chronological ordering of growth stages. The solid black arrows indicate the net motion of the tissue boundary, whereas the magenta arrows indicate the local tissue motion. The green shading in **a**–**d** demarcates the convergent extension phase of growth. **b**,**c**, Tissue-scale-observable fields in the germband of a single embryo. Tissue cartography ensures that the 3D geometry is properly handled. The shading corresponds to the two observed stages of growth. Mean cell density and total tissue area (**b**). The blue-shaded region shows the standard deviation. Tissue length measured along the A–P axis and the tissue width measured along the D–V axis (**c**). **d**, Mean residual value between the measured cell velocities extracted from a single embryo and the cell velocities predicted by the active hydrodynamic model. **e**, Magnitude of the velocity of the mean cell division event (190 division events extracted from a single embryo). The arrows showing the orientation are scaled by the norm of the velocity. **f**, Schematic of the flow field induced by a single division event. The magenta arrows indicate the local tissue flow. **g**–**i**, Cell velocities measured by single-cell tracking at three representative time points (76.2 h AEL (**g**), 82.4 h AEL (**h**), 91.1 h AEL (**i**)). **j**–**l**, Predicted cell velocities at the same times as **g**–**i**. The cyan lines in **g**–**l** indicate the cell velocities. **m**–**o**, Single-cell velocity residuals for the measured and calculated velocity fields shown in **g**–**i** and **j**–**l**, respectively.

**Fig. 5 |:**
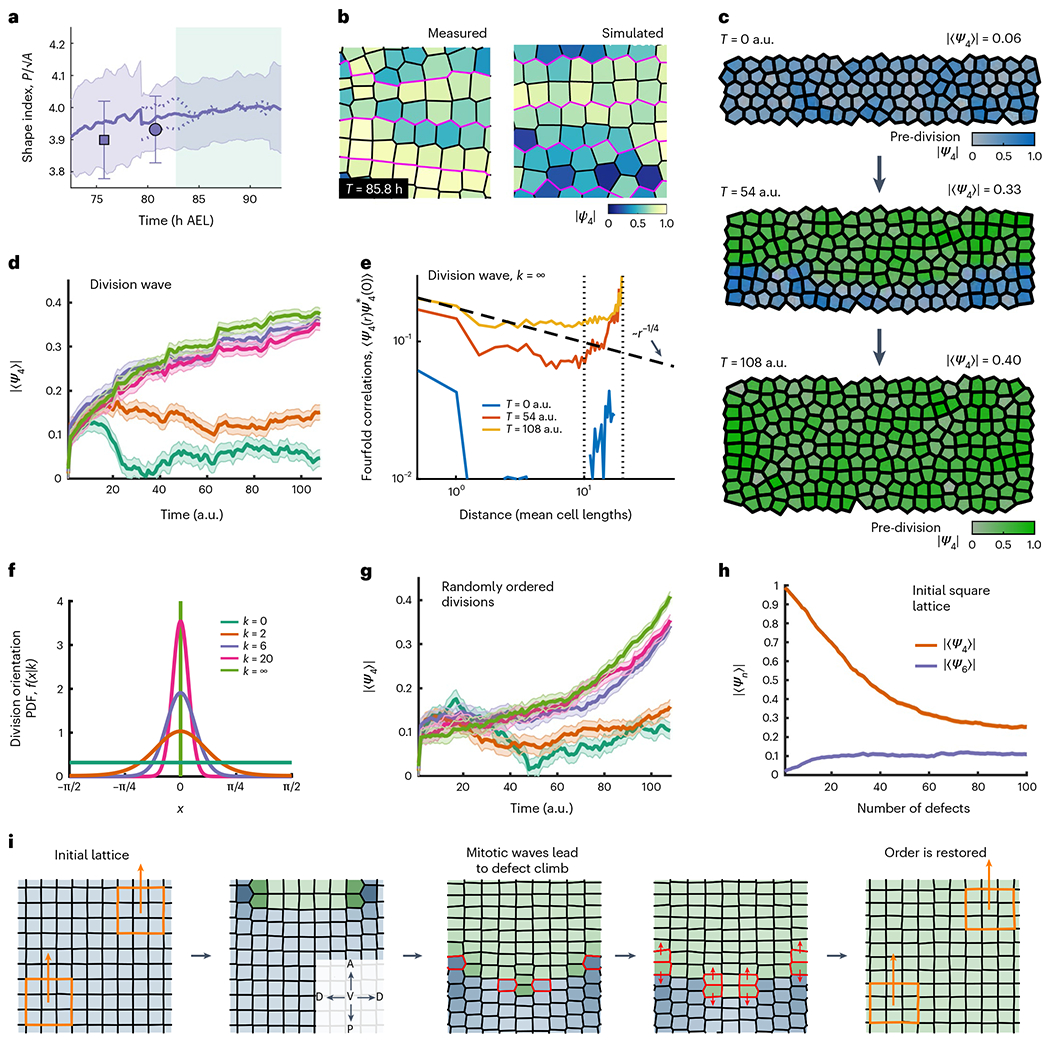
Oriented divisions generate fourfold order in vertex model simulations. **a**, Mean cell shape index $p_{0}=P/\sqrt{A}$ as a function of time in the trunk segmental ectoderm, where P is the cell perimeter and A is the cell area. The shaded region shows the standard deviation. Data are aggregated from four embryos. The dotted lines indicate the measured values from nuclear movies, whereas the markers indicate values measured from the snapshots of membrane marker movies (Methods). The error bars on the snapshot markers denote standard deviations. The shape index remains close to 4 (the shape index of a perfect square) for the entirety of ectodermal grid formation. The green-shaded region demarcates the convergent extension phase of growth. **b**, Comparison of cell geometry and fourfold order drawn from data (left) and simulation (right). The magenta lines denote the boundaries between adjacent parasegments. **c**, Illustration of a typical division wave simulation at three representative times (in arbitrary units). The hue corresponds to the number of cell divisions within a lineage and saturation represents the orientational order (disordered regions are less saturated). **d**, Absolute value of the mean fourfold order parameter during division wave simulations. Different curves correspond to different concentrations **k** of distributions from which division orientations were randomly drawn (shown in **f**). Above k≳5, the division wave reliably generates fourfold order. The reported values were averaged over five independent simulations for each value of k and the shaded regions show the standard error. **e**, Two-point correlation function of the fourfold orientational order parameter generated by division wave simulations with k=∞. The vertical lines indicate the length and width of the tissue. The final configurations exhibit quasi-long-ranged fourfold order. The dashed line illustrates algebraic decay of $∼r^{-1/4}$ and is intended as a guide for the eye. **f**, Illustrations of the von Mises distributions from which division orientations are drawn during simulation. Here k=0 corresponds to uniformly random divisions and k=∞ corresponds to perfectly oriented divisions. **g**, Absolute value of the mean fourfold order parameter generated using the same parameters as those in d, but with random division timings rather than a division wave. The reported values were averaged over five independent simulations for each value of k and the shaded regions show the standard error. **h**, Illustration of the breakdown of *n*-fold orientational order (*n* = 4, 6) in an initially square lattice due to the presence of defects inserted by cell divisions. **i**, Schematic summarizing the role of division choreography in generating and maintaining orientational order. The hue corresponds to the number of cell divisions within a lineage and the saturation and value represent the orientational order (less ordered regions are darker). The order is maintained and the grid is restored as long as the divisions are oriented along a single axis and each cell divides once before any particular cell divides twice. The cells divisions shown in the middle panels are highlighted in red. The orange regions in the first and last panels show that the direction of local order is coherent over long distances and preserved by the division choreography. In the inset in the second panel, A and P denote the anterior and posterior directions, whereas D and V denote the dorsal and ventral directions, respectively.

Since *Parhyale* is an emerging model organism, access to protocols and perturbations is sharply limited compared with established model organisms, such as *Drosophila* (ref. 21). A total of four embryos were used to generate the analysis in this work. Improvements to animal-handling techniques and imaging protocols are necessary for future work to involve larger sample sizes. Due to the challenges associated with the *in toto* live imaging of *Parhyale*, it was not possible to image all the embryos for the complete duration of germband extension. Two datasets featuring transgenic embryos with a fluorescent nuclear marker were produced. One embryo was imaged from 55.8 to 91.9hAEL, but only the period from 72.5 to 91.9hAEL was included in the analysis since the first part of the movie preceded germband extension. The validation of the hydrodynamic model (Fig. 4) was performed using this dataset. A second extended movie of a transgenic embryo with a fluorescent nuclear marker, filmed between 79.4 and 93.0hAEL, was also analysed and tracked. Other than the validation of the hydrodynamic model, all the major aspects of the analysis also drew from this dataset. Two movies featuring a lipid membrane dye FM-464 marker, rather than a nuclear marker, were also filmed between 75.8 and 79.4hAEL as well as 80.0 and 83.1hAEL. These movies were included in the analysis of the global bond-orientational order parameters (Fig. 2d) and cell shape index (Fig. 5a).

### Extraction of dynamical surfaces of interest

The output of the light-sheet microscope is a time series of 3D grids whose voxel values correspond to the intensity of the nuclear label or lipid dye. The extraction of the dynamical surface of interest from these datasets was performed in two stages: (1) 3D surface extraction and (2) 2D pullback map construction. In the surface extraction stage, the volumetric data of a representative time point were classified over the nuclear label/dye using the machine learning software called ilastik^43^. The resultant probability map was then fed into MATLAB R2019b and a static surface of interest was extracted using the morphological active contours method^44^ (Supplementary Fig. 1), a type of level-set-based segmentation algorithm well suited for segmenting complicated closed surfaces. The output of this segmentation is a 3D binary level set, with identical dimensions to the data, where ‘1’ values corresponded to the interior of the closed surface (all the embryonic tissues and yolk) and ‘0’ values corresponded to regions external to the *Parhyale* egg. The boundary of this binary level set is a point cloud, a subset of which included voxels corresponding to the embryonic tissue. This point cloud was subsequently triangulated using Poisson surface reconstruction^45^. The result was a topologically spherical mesh triangulation.

In the next processing step, this static surface was used as a seed to extract the dynamically changing surface at each time point. Recall that at this developmental stage, the embryonic tissue is a topological disc sitting on top of a spherical yolk. The embryonic tissue was, therefore, contained in a disc-like subregion of the sphere-like surface triangulation. To extract this region of interest, the entire sphere-like mesh was mapped into the plane using the orbifold Tutte embedding method^46^. This method generates a topologically consistent parameterization of the sphere in the plane, allowing us to view the entire surface at once with minimal geometric distortion. Next, a static submesh of the region of interest on the static surface was manually selected using the orbifold pullbacks. Although static, this region of interest was large enough that it contained all the relevant sections of the embryo as it grew and deformed over time. A set of ‘onion layers’ were then created by displacing the submesh along its positive and negative normal directions. A stack of pullback images were then created for each time point with one image in the stacks for each displaced onion layer. The number of layers and interlayer spacing were chosen so that all the geometric features of the dynamic surfaces were captured for the various time points somewhere within the image stack. These stacks were then fed back into ilastik and batch processed again over the nuclear label/dye. The result was a time-dependent field of normal displacements over the static seed surface that transformed the static surface into the corresponding dynamic surface for each time point. These dynamic triangulations of the evolving region of interest were then separately mapped into the unit disc conformally via Ricci flow^47^. Such a conformal mapping is only unique up to a Möbius automorphism of the unit disc. In other words, unless care is taken to register the pullbacks, the resultant images may be wildly misaligned in the pullback space from one time point to another. With this in mind, the time series of conformal pullbacks was iteratively registered to fix the conformal degrees of freedom within the pullbacks. Essentially, corresponding mesh vertices at subsequent times were approximately matched in two dimensions by finding an optimal Möbius automorphism of the unit disc that registered as many points as possible without sacrificing the conformality of parameterization^48^. The final result was a sequence of maximally aligned conformal pullbacks of the growing embryo to the plane. The conformality of these discrete parameterizations is illustrated in Supplementary Fig. 2.

It is worthwhile here to briefly discuss the constraints of visualizing curved surfaces in the plane via their parameterizations. Gauss’ celebrated Theorema Egregium forbids the construction of a globally isometric planar parameterization for a 2D surface with a non-zero Gaussian curvature^49^. In other words, if you want to map a curved surface into the plane, you can do so in a way that preserves angles (a conformal map), in a way that preserves areas (an authalic or isoareal map), or in a way that balances both non-zero angle distortion and non-zero area distortion, but you can never do so in a way that perfectly preserves the angles and areas everywhere. The central focus of this work is the orientational order of cells within a curved tissue. As such, we generally choose to visualize surfaces using conformal maps that preserve the angles between neighbouring cells from which the orientational order can be constructed. This means that some area distortion within the figures is inevitable. For instance, in Fig. 2c, it appears that lateral cells are larger than cells near the ventral midline. However, calculating the cell areas using tissue cartography reveals that the cell size is essentially uniform in the tissue at a given time point (Supplementary Fig. 13). Instead, the cell size primarily varies as a function of location in the cell cycle (recently divided cells are smaller; Supplementary Fig. 21b).

It should also be noted that the tissue cartography framework allows the user to implement not only conformal mappings but also a broad variety of parameterizations depending on the application. The visualization of the full embryo dynamics (Supplementary Video 4) was created using an ‘as-rigid-as-possible’ parameterization^50^. This parameterization attempts to produce a high-quality visual representation of the 3D surface in the plane by settling on an optimal balance of angle distortion and area distortion.

### In-plane cell segmentation and pathline reconstruction

One primary benefit of tissue cartography is that processing data in low dimensions greatly reduces the computational complexity of various analysis procedures. We exploited this benefit by directly segmenting the nuclei in the 2D pullback images. Images were first classified in ilastik. The resultant probability maps were then fed in MATLAB where the nuclei were segmented using a custom-built version of the watershed algorithm^51^. Custom additions to MATLAB’s built-in watershed functionality were necessary to account for spatially proximal nuclei that were initially undersegmented, that is, many nuclei were only counted as a single object. Special care was taken during this step in adjusting the watershed parameters to ensure that adjacent nuclei were properly distinguished from each other.

Once segmented, the nuclei were semiautomatically tracked using an enhanced point-matching procedure. For a given time point, the input to this procedure included a pullback image and segmentation at time t and another subsequent pullback image and segmentation at time t+1. First, the subsequent pullback image was registered onto the previous image using Demon’s deformable image registration algorithm^52^. The resultant displacement fields were then applied to the nuclei locations at time t+1. Point matching was then used to associate the nuclei locations at time t with the displaced nuclei t+1. Displacing the nuclei locations at t+1 to align more closely with the locations at t reduced the discrepancies in point matching associated with large nuclear motions. This process was iteratively applied until pathlines were generated for all the cell lineages at all the time points. Despite these enhancements, some manual correction was still necessary. These manual corrections were applied using a custom-built MATLAB graphical user interface. The pathlines were outputted as a digraph where the nodes represented particular cells at particular time points and edges stored the information about the temporal relationships between the nodes. The cell divisions could then be extracted from this tracking structure by locating events where single tracks split into two lineages.

Another benefit of tissue cartography is that the geometric information reflecting the fact that in-plane dynamics are occurring on a 2D surface embedded in 3D space are properly preserved. In particular, knowing the 2D locations of nuclei in pullback space provides an explicit correspondence to their locations on the surface in three dimensions. Therefore, once the tracks were constructed in two dimensions, it was trivial to extract full 3D nuclear pathlines. Velocities were constructed as simple backward differences between 3D nuclei locations. A backward difference was used since forward differences can generate ambiguities at cell division events where the forward difference velocities of the children sum to zero. The 3D velocities were decomposed into tangential and normal components relative to the dynamic surface. Tangential velocities were then consistently transformed back into the pullback space for display purposes using a discretization of the Jacobian on mesh triangulation faces.

### Region-of-interest selection for dynamical quantities

For any quantity depending on the measured cell tracking, with specific emphasis on the theoretical predictions of tissue velocities induced by cell divisions (Fig. 4), the region of interest was always defined to be the maximal set of PSPRs that we could accurately track and follow through at least one round of mitotic wave divisions. All other segments were either already substantially progressed into their second mitotic wave or had not completed their first mitotic wave by the end of our recordings. As time progresses, the region of interest expands to include all the clonal progeny of these chosen segments. The lateral extent of each PSPR was also carefully considered. It is understood that many cells at the dorsal boundary of the ventral ectoderm are in fact extra-embryonic and do not remain in the tissue as morphogenesis proceeds^22,25^. On these grounds, any cell near the dorsal boundary of the tissue that appeared to detach from the ectoderm or that could not be confidently associated with a particular PSPR was excluded from any subsequent analysis.

### Calculation of discrete curvatures

The discrete curvature was calculated for each dynamic mesh triangulation as a function of time using standard discrete constructions (Supplementary Fig. 24)^53^. The Gaussian curvature $K\left(v_{i}\right)$ of a mesh vertex $v_{i}$ was taken to be

(3) $$K\left(v_{i}\right)=\frac{1}{A_{i}}\left(2\pi -\sum_{F_{j}\in {𝒩}_{F}\left(v_{i}\right)}\theta_{F_{j}}\right),$$

where $A_{i}$ is the area associated to each vertex via the barycentric subdivision of the triangles attached to that vertex, ${𝒩}_{F}\left(v_{i}\right)$ is the set of incident faces $F_{j}$ attached to $v_{i}$ and $\theta_{F_{j}}$ is the internal angle of face $F_{j}$ corresponding to $v_{i}$. The mean curvature $H\left(v_{i}\right)$ of a vertex $v_{i}$ was calculated according to

(4) $$\Delta {\vec{x}}_{i}=2H\left(v_{i}\right){\hat{n}}_{i},$$

where ${\vec{x}}_{i}$ is the 3D location of vertex $v_{i},{\overset{ˆ}{n}}_{i}$ is the unit normal vector corresponding to vertex $v_{i}$ and Δ denotes the Laplace–Beltrami operator. The discrete Laplace–Beltrami operator was implemented using the familiar co-tangent discretization as^53^

(5) $$\Delta {\vec{x}}_{i}=\frac{1}{2A_{i}}\sum_{v_{j}\in {𝒩}_{v}\left(v_{i}\right)}\left(\text{cot}\;\alpha_{ij}+\text{cot}\;\beta_{ij}\right)\left({\vec{x}}_{j}-\vec{x_{i}}\right),$$

where ${𝒩}_{v}\left(v_{i}\right)$ is the neighbourhood of vertices attached to vertex $v_{i}$, and $\alpha_{ij}$ and $\beta_{ij}$ are the two internal angles of the triangles opposite the edge shared by vertex $v_{i}$ and $v_{j}$. To extract $H\left(v_{i}\right)$ from equation (4), it was first necessary to calculate ${\overset{ˆ}{n}}_{i}$, which was taken to be the angle-weighted average of the face unit normal vectors of the triangles incident to vertex $v_{i}$ assuming a counterclockwise orientation of vertices in the faces. This choice breaks the degeneracy in the orientation of the unit normal and allows for the simple extraction of a signed mean curvature. The panels in Supplementary Fig. 24 were constructed by averaging the Gaussian and mean curvatures of all the vertices found to lie within a particular Voronoi polygon corresponding to a specific cell after conformally mapping the dynamic meshes into the plane.

### Construction of correlation functions

To construct the two-point orientational order correlation functions, we first calculated the fourfold and sixfold orientational order parameters for each cell at a particular time point. Next, for each pair of cells, denoted here by their locations $x_{1}$ and $x_{2}$, we calculated the intercellular distance. This distance was taken to be the geodesic distance along the dynamic surface between the locations of the cell centroids on the 3D mesh triangulation^54^. We also calculated the product $\psi_{n}\left(x_{1}\right)\psi_{n}^{*}\left(x_{2}\right)$ for each pair of cells, where n∈{4,6}. We then partitioned the intercellular distances into a set of bins. All the pairs whose spacing lay between r and r+dr, where dr was the width of a bin, were then averaged together to calculate the two-point orientational order correlation function $<\psi_{n}\left(x_{1}\right)\psi_{n}^{*}\left(x_{2}\right)>$. For this calculation, it was assumed that this quantity only depended on the scalar distance r between the pairs of cells, that is, $\left\langle \psi_{n}\left(x_{1}\right)\psi_{n}^{*}\left(x_{2}\right)>=<\psi_{n}\left(r_{12}\right)\psi_{n}^{*}(0)>\right.$. Note that under averaging, only the real part of the product $\psi_{n}\left(x_{1}\right)\psi_{n}^{*}\left(x_{2}\right)$ contributed, since $\psi_{n}\left(x_{1}\right)\psi_{n}^{*}\left(x_{2}\right)+\psi_{n}\left(x_{2}\right)\psi_{n}^{*}\left(x_{1}\right)=2Re\left[\psi_{n}\left(x_{1}\right)\psi_{n}^{*}\left(x_{2}\right)\right]$. Finally, the intercellular distances were normalized by an average cell-length scale, calculated as the square root of the average area contained by each cell’s Voronoi polygon mapped back into three dimensions.

Following previous work^16,20^, information about translational order was extracted using variations in the pair correlation function g(Δr), that is, the high-resolution histogram of oriented geodesic distances between pairs of cells normalized by the number of pairs expected for a Poisson-distributed set of cell centres. Here an oriented geodesic distance $\Delta r_{12}$ from $x_{1}$ to $x_{2}$ consisted of a scalar geodesic distance $r_{12}$ and an orientation of the outgoing geodesic curve in the tangent space of the surface at $x_{1}$. Integrating this function over all the orientations of the separation vectors yields the isotropic pair correlation function g(r) (also known as the radial distribution function), which gives information about the positional order present in all the directions as a function of distance. In addition to g(r), we also constructed anisotropic distributions that only aggregated information about oriented pairwise separations along the A−P and D−V axes $\left(g_{AP}(r)\right.$ and $g_{DV}(r)$, respectively). The construction of all these distributions proceeded similar to the construction of the orientational order correlation function and also relied on the calculation of the pairwise geodesic distance between the cells. All the pairs of cells whose separation lay between r and r+dr, and whose relative orientation lay along the appropriate axes in the case of $g_{AP}(r)$ and $g_{DV}(r)$, were consolidated into histograms within a set of bins (of width dr) segmenting the intercellular spacing. The histograms were then normalized using an ‘effective volume method’ that directly accounts for and ameliorates finite size effects^55^. In the following explanation, we focus on the radially symmetric pair correlation function g(r) for simplicity. Naively, the pair correlation function is normalized by the expected number of cell pairs predicted by a Poisson distribution, that is

(6) $$g(r)=\sum_{i=1}^{N}\frac{n_{i}(r)}{N\rho \;\text{d}V},$$

where $n_{i}(r)$ is a count of the number of cells having their centres at a distance between r−dr/2 and r+dr/2 from the centre of the *i*th cell in the measurement volume, N is the total number of cells in the measurement volume, ρ=N/V is the raw number density of cells in the measurement volume, V is the total measurement volume and dV is the volume of the generalized shell between r−dr/2 and r+dr/2(dV=2πrdr for a 2D shell). Instead, we report the quantity as

(7) $$g(r)=\sum_{i=1}^{N}\frac{n_{i}(r)}{N\rho \;\text{d}V_{i}},$$

where now, $dV_{i}$ is defined as the intersection of the shell dV(r) centred on cell i with the finite measurement volume V. This intersection volume, therefore, had to be individually calculated for each cell and for each distance bin. The final reported quantities were g(r)−1 since this quantity decays to zero in a disordered system. An analysis of the finite size effects present in both orientational and translational correlation functions can be found in Supplementary Fig. 9.

### Circular statistics for division events

To properly analyse the distributions of division events, it was necessary to construct the measures of statistical properties that properly accounted for the nematic nature of division events, that is, a division event with orientation θ is physically identical to a division event with orientation θ±π. Extending familiar measures of circular distributions^56^, the modified circular mean of the orientations of a set of division events, $\left\{\theta_{n}\right\}$, where n=1,…,N, was defined to be

(8) $$\bar{\theta }=\frac{1}{2}\text{arg}\;\left[\sum_{n=1}^{N}{\text{e}}^{2\text{i}\theta_{n}}\right]\in \left[-\frac{\pi }{2},\frac{\pi }{2}\right],$$

which is invariant under the transformation $\theta_{n}\to \theta_{n}\pm \pi$ for any $\theta_{n}$. If the division orientations are tightly distributed around a single value, then this quantity will also be close to that particular value. We also define a modified measure of angular dispersion as

(9) $$s=\sqrt{2\left(1-\frac{1}{N}\sum_{n=1}^{N}{\text{e}}^{2\text{i}\theta_{n}}\right)}.$$

Note that this is a dimensionless quantity. Our measure of angular dispersion varies between s=0 for perfectly oriented divisions $\left(\theta_{n}=\overset{¯}{\theta }\right.$ for all *n*) and $s=\sqrt{2}$ for totally isotropic divisions.

### Determination of average division velocity

Division events were extracted from the tracking structure and the resulting sample set was pruned for quality (sufficiently far from the boundary of the tissue, sufficiently far from another division event and so on). To compare different events, divisions were translated and rotated so that the centre of mass of the daughter cells lay at the origin and the division axis lay along the *y* axis. The measured velocities of the daughter cells and cells in the third-order natural neighbourhood of the daughter cells were then interpolated onto fine-mesh triangulation using generalized Hessian energy scheme that minimizes distortion in the interpolated field at the boundary of the triangulation^57^. The interpolated velocity fields were then averaged across division events to find the mean velocity induced by divisions (Fig. 4e and Supplementary Fig. 14). Gradients of the resultant velocity fields were calculated on the mesh triangulation using a custom-built implementation of the discrete exterior calculus^58^ in MATLAB (Supplementary Fig. 15).

### Numerical prediction of tissue velocities from cell divisions

An active hydrodynamic model was used to predict the tissue velocities resulting from the collective motion induced by cell divisions. We briefly explain the model here to properly describe how it was numerically solved to predict the tissue velocities using actual data. The full details of the model, including a derivation, are presented in the Supplementary Text. Tissue velocities were calculated as the solution to the following boundary value problem:

(10) $$v_{1}\nabla^{2}v+v_{2}\nabla (\nabla \cdot v)=-\nabla \cdot \sigma^{a}\equiv -F^{a},$$

where v denotes the tissue velocities; $\sigma^{a}$ are the active stresses due to divisions; $F^{a}$ are the corresponding active forces; $v_{1}=\tau_{R}\mu$ is the effective shear viscosity; $v_{2}=\tau_{R}(\mu +\lambda )$ is the effective bulk viscosity; $\tau_{R}$ is a timescale corresponding to mechanical relaxation due to growth/tissue remodelling; and μ and λ are the familiar Lamé parameters of continuum elasticity. To simplify the numerical analysis, this model was reformulated as

(11) $$\nabla^{2}v+\frac{1}{1-2v}\nabla (\nabla \cdot v)=-{\tilde{F}}^{a},$$

where now ${\tilde{F}}^{a}$ is a renormalized set of active forces and v is analogous to the Poisson ratio obtained by treating the tissue as an idealized thin 3D material, that is, as opposed to a true 2D material. The (renormalized) active forces induced by each division event were modelled as resulting from a circular Eshelby inclusion of radius a and eigenstrain

(12) $$\epsilon^{*}=(M/2)I+q(2\hat{n}⊗\hat{n}-I),$$

where I is the 2×2 identity matrix and $\overset{ˆ}{n}$ is a unit vector along the division axis. Here M is a parameter controlling the isotropic contribution of the divisions that mediates area growth and q controls the deviatoric contribution that mediates constant-area shear.

The model was numerically solved for actual data using custom finite-element-method machinery. First, a fine-mesh triangulation was constructed over a subset of tracked cells at a particular time (Supplementary Fig. 16a). Circular holes containing cells about to divide were then removed from the triangulation. These holes represented the finite-sized circular Eshelby inclusions used to model the velocities induced by cell divisions (Supplementary Text). The size of the holes was chosen so that the area of the circular holes equalled the area of the Voronoi polygons of the corresponding cells. Removing the inclusions from the mesh renders the task of predicting velocities a simple boundary value problem with Dirichlet boundary conditions. The velocities on the interior boundary vertices were set to match the analytical predictions of the model (that is, the displacement field induced by the inclusion) plus a constant term equal to the average measured velocity of the entire tissue to account for the advection of the dividing cell with the tissue-scale flow. This choice constrained simultaneous inclusions to move with the same advection velocity. In principle, one might expect that each inclusion should advect with its own velocity, which could be derived from the data, for instance, by averaging the measured velocities in a local region around each division event. Numerical experiments, however, show that this additional machinery produces virtually indistinguishable results from the uniform advection velocity implementation. This insensitivity can be understood by noting that the velocity induced by each division event dwarfed the bulk velocity of the rest of the tissue $\left(V_{div}/<V_{bulk}>\approx 7\right)$, such that minor changes to the imposed advection velocity did not strongly alter the results. The velocities on the exterior boundary were set to the measured velocities. Using the measured velocities on the exterior boundary captured how the cells not included in the subregion contributed to the relevant motion within the region over which the velocities were predicted. The results of the numerical solution were a velocity vector for each triangulation vertex. For cells that did not divide, all the vertices contained within each corresponding Voronoi polygon were then averaged to produce a single-cell velocity vector. Note that many vertices were averaged for each polygon, so that prescribing Dirichlet boundary conditions on the triangulation did not correspond to prescribing cell-scale velocities. Note that under deformation, the circular inclusions were deformed into ellipses. The locations of the foci of these ellipses were directly given by the fluid mechanical model. The velocity of cells that divided were set to be the displacement of these foci from the centre of the undeformed circular inclusion. For our purposes, we set v=1/3 for all the time points and parameters M and q were separately fit for each time point containing divisions using MATLAB’s lsqnonlin to minimize the resulting velocity residuals. To produce the ‘division-only’ predictions (Supplementary Fig. 18), we simply summed the contribution of the analytical prediction for each division event at each time point without solving the boundary value problem using the measured data on the domain boundary.

### The velocity residual

To compare the measured flow fields v(x) with the predicted flow fields u(x) in a quantitative fashion, we defined a global measure for the spatial velocity residual that was insensitive to noise-dominated fluctuations in the regions of slow flow. Let

(13) $$<u>\equiv \sqrt{{<u(x)\cdot u(x)>}_{\text{embryo}}}$$

define an overall magnitude of the field u(x). Here $<u(x)\cdot u(x){>}_{embryo}$ denotes an average of the spatially dependent field $u^{2}(x)=u(x)\cdot u(x)$ over the entire embryo and is, therefore, not space dependent. We define our velocity residual as

(14) $$R=\frac{\left(<u{>}^{2}v^{2}(x)+u{\left(x\right)}^{2}<v{>}^{2}\right)-2\sqrt{<u{>}^{2}<v{>}^{2}}v(x)\cdot u(x)}{2<u{>}^{2}<v{>}^{2}}.$$

This residual provides a spatial discrepancy map, quantifying the prediction quality as a function of location on the embryo. An identical velocity residual was used elsewhere^33^.

### Generation of synthetic datasets and comparison with measured data

The synthetic datasets were generated to understand the extent to which the measured order was statistically significant and how the finite size of the germband affected the order. First, the length and width of the germband at each time was extracted. These were determined by tagging the representative rows and columns of cells, following these rows and columns over time and calculating the geodesic length along each row and column. The average geodesic length of the rows (columns) was taken to be the width (length) of the germband at a particular time. We also extracted a mean cell density at each time point by averaging the inverse 3D areas of cells. Here 1,000 synthetic datasets were generated for each time point within rectangles with the same length and width as the germband. Points representing the cell centroids were generated to exhibit the same cell density as the germband using a fast Poisson disc sampling method^59^. The connectivity of these randomly generated points was approximated using Voronoi tesselation. This connectivity allowed for the calculation of the order parameters and orientational correlation functions over the finite-sized samples. We also calculated the radial distribution functions along the A−P axis (length of the rectangle) and D−V axis (width of the rectangle) for each synthetic dataset (Supplementary Fig. 8) using the same method of construction as the measured data.

Additionally, we compared the corresponding measured and synthetic distributions of orientational order parameters according to the two-sample Kolmogorov–Smirnov (K−S) test^60^ using MATLAB’s kstest2. This implementation of the K−S test returns two measurements that assess the confidence with which one can assert that two sets of observed random variables are drawn from the same distribution. The first is the K−S statistic, which is simply the maximum difference between the empirical cumulative distribution functions of the two sample sets. The larger the K−S statistic, the greater is the discrepancy between the two sample sets. The second measurement is the asymptotic p value, which is the probability of observing a test statistic as extreme as–or more extreme than–the observed value under the null hypothesis that both samples are drawn from the same distribution. All the K–S statistics and p values are reported in Supplementary Table 1. At the confidence level of α=0.05, the null hypothesis is rejected for all the distributions, indicating it is unlikely that the observed order at any time point is due to chance. We note that the p values for fourfold order at intermediate and late times are vastly smaller than the corresponding p values at early times, whereas the p values for sixfold order do not change as drastically. This implies that although it is still improbable that the observed fourfold order at early times is due to chance, it is hugely more likely compared with intermediate and late times after the order-generating choreography has unfolded.

Similar methods were used to generate the synthetic illustration of a translationally ordered system (Supplementary Fig. 7). We patterned perfect square lattices on rectangles with the same length and width as the germband for the same representative time points. The lattice spacing was set to match the measured cell density at the corresponding time point. We then generated 100 synthetic datasets for each time point by adding Gaussian white noise to the lattice site positions with a signal-to-noise ratio of 5. The calculation of the orientational order parameters, orientational correlation functions and radial distribution functions were performed in the same manner as the other synthetic datasets.

### Reporting summary

Further information on research design is available in the Nature Portfolio Reporting Summary linked to this article.

## Supplementary Material

SI

SI Video 1

SI Video 2

SI Video 3

SI Video 7

SI Video 8

SI Video 6

SI Video 4

SI Video 9

SI Video 10

SI Video 5

## Figures and Tables

**Fig. 1 |:**
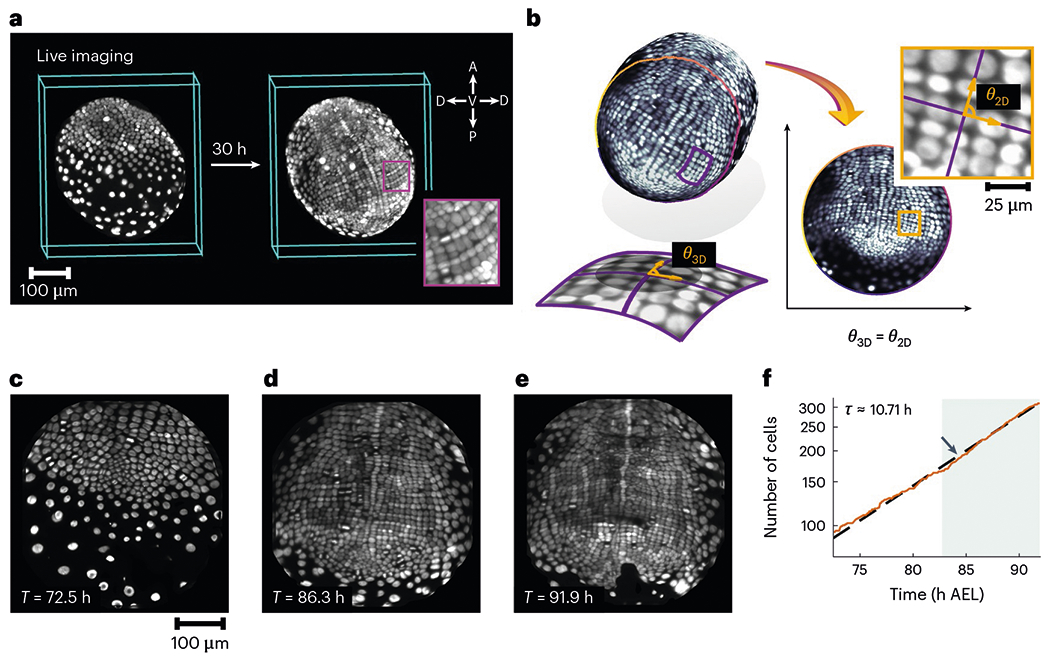
Conformal tissue cartography captures cell and tissue geometry in vivo. **a**, Multiview light-sheet microscopy allows for the non-invasive 3D imaging of growing *P. hawaiensis* embryos. The symbols A and P in the inset denote the anterior and posterior directions, whereas D and V denote the dorsal and ventral directions, respectively. **b**, Conformal tissue cartography faithfully captures the relative orientations of cells and streamlines the data analysis via dimensional reduction. Here $\theta_{3D}$ denotes the angle between two curves on the 3D surface (shown in purple), whereas $\theta_{2D}$ denotes the angle between the image of the same two curves in the 2D domain of parameterization. **c**–**e**, Trunk ectodermal germband pulled back to the plane by tissue cartography at 72.5 h AEL (**c**), 86.3 h AEL (**d**) and 91.9 h AEL (**e**). **f**, Number of cells in a tracked region within the tissue, comprising four parasegments in a single embryo, shown on a log scale. The dashed line is an exponential fit to the cell-doubling time *τ*. The shaded region highlights the convergent extension phase of growth (Fig. 4).

**Fig. 3 |:**
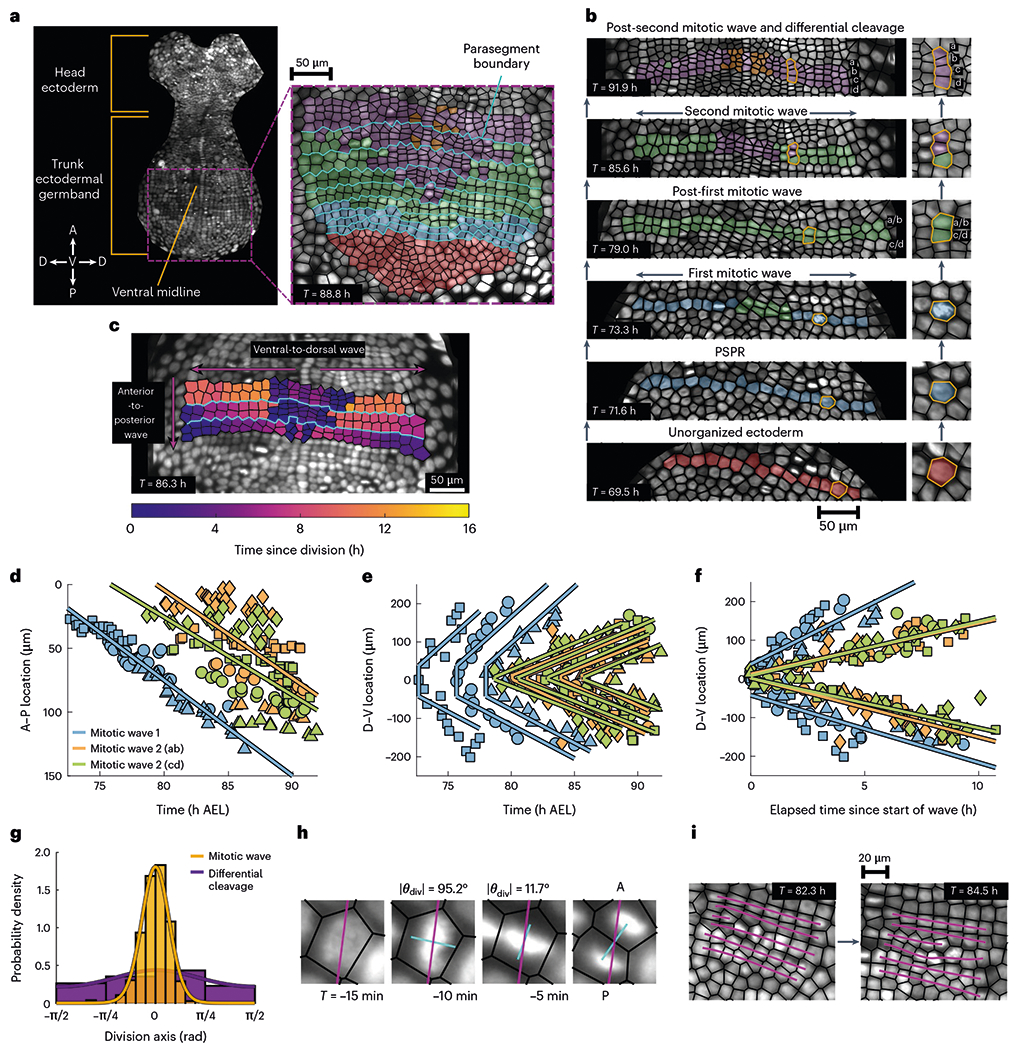
Waves of actively oriented divisions generate fourfold order. **a**,**b**, Schematic of parasegment formation and cell proliferation in the trunk ectodermal germband. In **a** (left), A and P denote the anterior and posterior directions, whereas D and V denote the dorsal and ventral directions, respectively. The head ectoderm and trunk ectoderm are demarcated and the ventral midline is highlighted, too. Also, **a** (right) shows the spatial distribution of cell populations delineated by the number of divisions after parasegment formation once parasegments are assembled from the pool of unorganized ectoderm at the posterior pole. The cyan lines demarcate the parasegment boundaries. Proliferation of a single parasegment over the course of 22.4 h (**b**). Initially unorganized ectoderm at 69.5 h AEL coalesce into a PSPR by 71.6 h AEL. Cells comprising the PSPR then divide along the A−P axis as part of a mitotic wave, shown partially completed at 73.3 h AEL, which originates at the ventral midline and spreads outwards until there are two complete rows of cells (79.0 h AEL), denoted as ‘a/b’ and ‘c/d’. Each of these two rows then undergoes a second mitotic wave, shown partially complete at 85.6 h AEL, until there are four complete rows, denoted as ‘a’, ‘b’, ‘c’ and ‘d’, reflecting the origin of each row from its predecessor at the two-row stage. After the second mitotic wave, rapid differential cleavage sets in near the ventral midline, in which cells divide isotropically (91.9 h AEL). The insets (right) highlight the behaviour of a single cell and its progeny throughout the mitotic waves. **c**, Snapshot of elapsed time since division reveals two orthogonal phase waves within and across parasegments. The cyan lines demarcate the parasegment boundaries. **d**–**f**, Location of mitotic wave division events over time for a single embryo. The shapes indicate the parasegment within which a division occurs. The indicated lines are linear fits to all the division events associated to a particular mitotic wave. The linear fits of divisions within a single parasegment on either side of the ventral midline in e are joined by a vertical line as a guide for the eye. Location of each division along the A−P axis (**d**). The speed of mitotic wave 1 is $7.5\pm 0.3\mu m\;h^{-1}$. The speed of mitotic wave 2(AB) is $6.9\pm 0.9\mu m\;h^{-1}$. The speed of mitotic wave 2(CD) is $6.1\pm 0.9\mu m\;h^{-1}$. Also, **e** and **f** show the location of each division along the D−V axis. The division times in **f** have been normalized to the occurrence of the first division event associated with a particular wave in a specific parasegment. The speed of mitotic wave 1 is $19.2\pm 2.1\mu m\;h^{-1}$. The speed of mitotic wave 2(AB) is $13.9\pm 1.2\mu m\;h^{-1}$. The speed of mitotic wave 2(CD) is $13.1\pm 0.9\mu m\;h^{-1}$. The analysis of division locations and timings for a second embryo is shown in Supplementary Fig.11. **g**, Orientation of cell division axes relative to the A–P axis. The histogram includes 483 mitotic wave divisions and 112 differential cleavage divisions from two embryos. The indicated curves are von Mises distributions fit to histogram counts. The circular mean division angle and angular deviation for the mitotic waves are ${\overset{‾}{\theta }}_{MW}=0$ rad and $s_{MW}=0.44$. The circular mean division angle and angular deviation for the differential cleavage are ${\overset{‾}{\theta }}_{DC}=0.04rad$ and $s_{Mw}=1.29.h$, Example of active reorientation of a nucleus immediately before cell division. Here $\theta_{div}$ denotes the angle between the A–P axis (magenta lines) and the presumptive division axis (cyan lines). The cyan line in the final image indicates the actual division axis. **i**, Schematic of division-induced defect climb along parasegments between 82.3 and 84.5 h AEL. The magenta lines indicate the cell-row identity and are intended as guides for the eye to highlight the time evolution of the defect.

## Data Availability

The 2D pullback images and 3D surface mesh triangulations for all the time points of all the datasets and the simulated data used for order parameter measurement validation are available via Dryad at https://doi.org/10.1101/2021.07.28.453899 (ref. 61). Raw light-sheet microscopy output is available from the corresponding authors upon request (D.J.C. or S.J.S.).
