# Supplementary material for: Active cell divisions generate fourfold orientationally ordered phase in living tissue
Source: Nat Phys. Author manuscript; Available in PMC 2023 Oct 2. (PMC10545346; doi:10.1038/s41567-023-02025-3)
Supplement: SI [file NIHMS1900676-supplement-SI.pdf]

nature physics

Article https://doi.org/10.1038/s41567-023-02025-3

Active cell divisions generate fourfold orientationally ordered phase in living tissue

In the format provided by the authors and unedited

Supplementary Text

Simulation of the vertex model

We simulate a simple vertex model with energy

$$E = \sum_c \left[\frac{k_{A,c}}{2} (A_c - A_{0,c})^2 + \frac{k_{P,c}}{2} (P_c - P_{0,c})^2 \right], \tag{1}$$

where c labels the cells, $k_{A,c}$ is the area stiffness parameter for cell c, A_c is the cell area, $A_{0,c}$ is the target cell area, $k_{P,c}$ is the perimeter stiffness parameter, P_c is the cell perimeter, and $P_{0,c}$ is the target cell perimeter. The first term in the sum is an elastic energy that tries to force each cells area to match its target area and the second term is a similar contribution that tries to force each cell to obtain a specified target perimeter. The code used to simulate the vertex model was based on the publicly available code used in [1]. For simplicity, in all of our simulations, we set $k_{A,c} = k_{P,c} = 1$ and fix the target areas, $A_{0,c} = A_0$, and perimeters, $P_{0,c} = P_0$, to be uniform for all cells yielding the simplified energy

$$E = \frac{1}{2} \sum_c \left[(A_c - A_0)^2 + (P_c - P_0)^2 \right]. \tag{2}$$

The simulation algorithm is detailed in Algorithm 1.

Initial lattice construction

For the division wave/random division simulations, the initial lattices were generated by first constructing a perfect hexagonal lattice with a specified length and height. Row identities were extracted from this initial stage and a subset of the rows in the middle of the lattice were selected to be a part of the division order generated using the row identities. For the random division simulations, this initial division order was then randomly permuted. All simulations were run with an initial set of five dividing rows. Gaussian white noise with a signal-to-noise ratio of 18 was then added to cell positions to produce a nonuniform hexatically ordered initial state (i.e. quasi-long range sixfold orientational order and no translational order). The decision to use a hexatically ordered initial state was due to the necessity of having an initial configuration with well defined rows and with no fourfold orientational order. Properties of the initial lattices are displayed in Fig. S19. After the initial lattice was constructed, the parameter A_0 was set to be the mean area of all the cells in the lattice and $P_0 = 4A_0$. Lattices were constructed so that all cells had an approximate initial length scale $= 1$.

Growth, division, relaxation, and confinement

After the initial lattice was constructed, simulations begin with the first division wave. The cell that is next to divide in the division order undergoes a series of growth steps. In each growth step, the parameters $P_{0,c}$ and $A_{0,c}$ were increased, fixing $P_{0,c} = 4A_{0,c}$. Lattice connectivity was then updated by implementing T1 transitions for all edges below a user

Algorithm 1 Vertex Model Simulation Algorithm

Require: Initial cell lattice, initial division order for first wave

```
Relax initial cell lattice
for Each division wave do
    Update division order for current wave
    for Each cell in the division order do
        for Number of growth steps do
            Update A_0, P_0 for the next cell in the division order
            Update mesh connectivity due to T1 transitions
            Relax cell lattice
            Enforce confinement
        end for
        Re-set A_0, P_0 for the cell about to divide
        Update mesh connectivity due to T1 transitions
        Randomly choose division axis orientation
        Divide cell along division axis
        Relax cell lattice
        Enforce confinement
    end for
end for
```

specified length threshold. Boundary edges whose shared external angle dropped below a user specified threshold were then fused together to prevent self-interpenetration of the tissue and other physically erroneous configurations. For all of our simulations, we set the T1 edge length threshold $\ell_{min} = 0.05$, the edge merger threshold $\theta_{min} = 20^{\circ}$, and the number of growth steps $N_{growth} = 50$. The geometric parameters $P_{0,c}$ and $A_{0,c}$ were increased so that $A_{0,c} = 1.5\, A_0$ after N_{growth} growth steps. Additionally, for all of our numerical experiments T1 transitions were prohibited in the bulk.

After all parameter updates and topological corrections, the lattice was then relaxed to an equilibrium configuration. The energy was minimized with the GNU Scientific Library Multidimensional Minimizer, using the Polak-Ribiere conjugate gradient algorithm. The wrapper to this minimization procedure was extended from the code used in [1]. Following relaxation, confinement was enforced by clipping the x-coordinates of lattice vertices to remain within a user specified domain, equal to the width of the original lattice domain plus one cell length. This choice was consistent with the observed behavior of the germband during the first stage of growth (Fig. 4C).Using a large number of growth steps ensured that no more complicated constraint enforcement procedure was necessary.

After all of the growth steps were concluded, the cell was then forced to divide. Division orientations were drawn from a circular von Mises distribution given by

$$f(x|\mu, k) = \frac{1}{\pi I_0(k)}\, e^{k\,\cos(2(x-\mu))}, \tag{3}$$

where μ is the center of the distribution, k is its concentration, and $I_0(k)$ is a modified Bessel function of the first kind. We set $\mu = \pi/2$ for all of our simulations, i.e. a vertically oriented division along the A-P axis. See Fig. 5F for a visual depiction of the distributions for all values of k used in the simulations. Following division, the tissue was relaxed and confined again and the whole procedure continued for the next cell in the division order.

Once all of the cells in the division order had divided, the division order was updated for the subsequent wave so that the new order would also proceed as a division wave. We only simulated a single division wave in our numerical experiments. Visualization and order parameter calculations were performed on the Voronoi tessellation of the centroids of each cell polygon in order to be consistent with the methodology used to analyze the data.

Fluid mechanical model

We investigated the role of cell divisions in producing tissue scale flow fields through the use of an active hydrodynamic model. Continuum mechanics is an effective method for capturing how force balance is associated with local deformation and flow [2]. In the simplest models, the forcing that generates flow can be neatly decomposed into a set of separate passive and active contributions. The tendency of cells to resist deformation and the viscous coupling between cells translate local forces into long range flows. Interactions among these various contributions, both locally and over large scales, can be then be built up with increasing degrees of complexity to create more accurate descriptions of biological realities. This framework has proved effective in numerous biological applications elucidating how active forces generate non-trivial flow fields [3–5].

The tissue scale mechanics of epithelia is best understood as emerging from the collective mechanics of the tissue's constituent cells [6]. The cell scale mechanics are dominated by the influence of intracellular cytoskeletal cortices [7, 8]. These structures are capable of supporting stresses throughout the cell's bulk interior and can deform the cell either actively or in response to external stresses. The individual cortices of neighboring cells are coupled together by cadherin mediated adherens junctions [9] into a global trans-cellular mechanical network. Crucially, these networks are both 'active' and 'adaptive'. Active contributions, such as cell divisions [10] or the contraction of trans-cellular actomyosin networks [4], act both locally and over large scales to deform the tissue. These active deformations generate passive stress distally from the activity via the coupling between cells. Stresses created in this fashion can then be relaxed either locally, via internal rearrangement of the cytoskeletal cortex [11, 12], or at the tissue scale, via cell rearrangements [3]. The result is an exotic type of viscoelasticity, wherein the tissue responds elastically to active stresses over short timescales and then reverts to a fluidlike flow at longer timescales as the tissue adaptively relaxes stress. When the timescale of growth (i.e. rate of cell division, etc.) is long compared to the timescale of mechanical relaxation, the tissue essentially behaves like a slowly creeping fluid in quasistatic mechanical equilibrium with both the active and external forces. For our purposes, a detailed description of the complex cellular processes mediating the active stresses and adaptive relaxation is unimportant. Instead, we adopt an approximate continuum scale description of the tissue that captures the relevant behaviors of the system.

At short time scales, the tissue behaves elastically [13, 14]. Incremental increases in

strain generate corresponding increases in stress. These stresses are then subsequently relaxed over a time scale τ_R. The total stress $\boldsymbol{\sigma} = \boldsymbol{\sigma}^e + \boldsymbol{\sigma}^a$ can be decomposed into a sum of the elastic stresses $\boldsymbol{\sigma}^e$ and the active stresses $\boldsymbol{\sigma}^a$. For simplicity, we assume that the tissue is a flat two-dimensional surface. Supposing the tissue behaves like a Maxwell viscoelastic fluid, the time-evolution of the elastic stress is given by

$$\dot{\boldsymbol{\sigma}}^e = \mu\left(\nabla\mathbf{v} + (\nabla\mathbf{v})^T\right) + \lambda\left(\nabla\cdot\mathbf{v}\right)\mathbb{I} - \tau_R^{-1}\,\boldsymbol{\sigma}^e, \tag{4}$$

where $\dot{\boldsymbol{\sigma}}^e = \partial_t\boldsymbol{\sigma}^e + \mathbf{v}\cdot\nabla\boldsymbol{\sigma}^e$ denotes a convective time derivative, $\mathbf{v}$ is the local velocity or rate of displacement, and μ and λ are the Lamé parameters characterizing a linear isotropic stress-strain relationship. The Lamé parameters are assumed to be spatially homogeneous, but may depend on time. A more complete description would include the corotational time derivatives of $\boldsymbol{\sigma}^e$ on the left hand side, but we omit them for simplicity. The first two terms on the right hand side describe the generation of stress in proportion to the rate of strain. The final term parameterizes the relaxation of the elastic stress. We have made the assumption that $\dot{\boldsymbol{\sigma}}^e$ only depends on $\boldsymbol{\sigma}^a$ implicitly through the final term $\tau_R^{-1}\boldsymbol{\sigma}^e$ (see the force balance condition below). This is not meant to imply that adaptive cell behaviors do not actually depend explicitly on the active stresses. It is merely a mathematical simplification. In reality, there may exist a complex interplay between mechanics, gene expression/cell fate, and adaptive cellular behaviors, but this lies beyond the scope of this work.

In general, the tissue flow velocity can be described by the Cauchy momentum equation [2]

$$\begin{aligned}\rho\,\dot{\mathbf{v}} &= \nabla\cdot\boldsymbol{\sigma} + \mathbf{F}^{EXT}\\ &= \nabla\cdot\boldsymbol{\sigma}^e + \nabla\cdot\boldsymbol{\sigma}^a + \mathbf{F}^{EXT},\end{aligned} \tag{5}$$

where ρ denotes the material density and $\mathbf{F}^{EXT}$ denotes any external body forces acting on the system. If the rate of mechanical relaxation is sufficiently fast to reach quasi-equilibrium, $\dot{\boldsymbol{\sigma}}^e$ vanishes, leaving us with

$$\tau_R^{-1}\boldsymbol{\sigma}^e \approx \mu\left(\nabla\mathbf{v} + (\nabla\mathbf{v})^T\right) + \lambda\left(\nabla\cdot\mathbf{v}\right)\mathbb{I}. \tag{6}$$

Substituting this into Eq. (5), we find that

$$\rho\,\dot{\mathbf{v}} = \tau_R\,\mu\,\nabla^2\mathbf{v} + \tau_R\left(\mu+\lambda\right)\nabla(\nabla\cdot\mathbf{v}) + \nabla\cdot\boldsymbol{\sigma}^a + \mathbf{F}^{EXT}. \tag{7}$$

In other words, the transient elasticity of the system gives rise to fluidlike behavior in the quasi-stationary regime parameterized by a set of effective viscosities. The effective shear viscosity $\nu_1 \equiv \tau_R\,\mu$ tends to resist shearing motion, while the effective bulk viscosity $\nu_2 \equiv \tau_R\left(\mu+\lambda\right)$ resists the isotropic compressible part of the flow. If the effective viscosities are sufficiently large and the corresponding motion is sufficiently slow we can neglect the inertial term relative to the viscous terms ($\rho\dot{\mathbf{v}} << \nu\nabla^2\mathbf{v}$). If, for simplicity, we furthermore assume that there are no external forces acting on the system, we are left with the following force balance condition defining the tissue flow velocities in terms of the active stresses

$$\nu_1\nabla^2\mathbf{v} + \nu_2\nabla(\nabla\cdot\mathbf{v}) = -\nabla\cdot\boldsymbol{\sigma}^a \equiv -\mathbf{F}^a, \tag{8}$$

which can be solved given suitable boundary conditions.

Cell divisions and active stresses

In general, there may be numerous different types of active stresses acting within the tissue during growth [4, 10, 15]. These various sources of active stress will likely not contribute with equal importance to the shaping of the nascent tissue. Our observations of the cell division choreography indicate that cell proliferation is the primary factor mediating tissue velocities. In order to simplify our model, we assume that the only relevant active stresses are due to these cell divisions.

Our first consideration in modeling active stresses due to cell divisions is to understand how division events might be incorporated into a spatiotempoally coarse grained scheme. With respect to timing, daughter cell separation in real cell division events occurs over a short, but finite time [16]. We ignore the complexity of the rapid subcellular properties mediating mitosis and model cell divisions as instantaneous events (for our purposes any process that occurs faster than 5 min, the time resolution of our microscope data, is considered instantaneous). Real cell divisions push on the surrounding tissue, which responds elastically over the short time scale of the actual division, before remodeling to relax the resulting stress. In our model, both the plastic strain due to a division and the subsequent relaxation are assumed to occur instantaneously so that the fluid is always in quasistatic mechanical equilibrium. With respect to spatial coarse graining, we note that the length scale of a single cell division is small compared to the scale of the entire germband. Numerical experimentation showed that approximating division events as point force dipoles produced inaccurate flow fields. We therefore model divisions as small, but finite size events to regularize this inconsistency. We also make the approximation that the inclusion lives in an infinite, otherwise quiescent medium in order to make analytic progress.

The mathematical structure of the fluid mechanical equations of motion in Eq. (8) is identical to the structure of the Navier equations of linear elasticity. Exploiting this similarity, we can model the instantaneous displacement (read velocity) due to a cell division as that of an Eshelby inclusion. In the context of classical elasticity, an Eshelby inclusion is a finite subregion of an elastic body which undergoes a permanent plastic deformation [17]. Eshelby inclusions, and the associated theories of elastic multipoles [18], have proved useful in numerous applications including understanding shear localization in amporphous solids [19] and the properties of mechanical metamaterials [20]. In the current context of viscoelastic tissue growth, this displacement corresponds to both the plastic deformation and the subsequent viscous relaxation, which are assumed to both occur instantaneously on time scales relevant to growth. Relying on the well-trodden mathematical history of Eshelby inclusions, we may, in the following derivation, use terminology similar to Eshelby's original exposition for elastic materials (e.g. eigenstrain). For clarity, we emphasize again that we are modeling the tissue as a viscoelastic fluid, not an elastic solid, and that the parameters of our model are effective viscosities, not elastic moduli which would vanish in an orientationally ordered phase.

In particular, we choose to model the division as a circular Eshelby inclusion of radius a and eigenstrain $\epsilon^*_{\alpha\beta} = (M/2)\,\delta_{\alpha\beta} + q\,(2\hat{n}_\alpha \hat{n}_\beta - \delta_{\alpha\beta})$, where $\delta_{\alpha\beta}$ is the Kronecker delta and $\hat{\mathbf{n}}$ is a unit vector along the division axis. Note that we have adopted an index notation to better account for the high-rank tensorial nature of the following calculations. Greek indices vary in the set $\{x, y\}$. We also adopt the Einstein summation notation so that

all repeated indices are summed. It was shown by Eshelby that the constraining medium generates a constant strain within the inclusion $\epsilon^c_{\alpha\beta} = S_{\alpha\beta\gamma\delta}\,\epsilon^*_{\gamma\delta}$, where $S_{\alpha\beta\gamma\delta}$ is a constant tensor for any elliptical inclusion. For a circular inclusion

$$S_{\alpha\beta\gamma\delta} = \frac{\lambda-\mu}{4(\lambda+2\mu)}\,\delta_{\alpha\beta}\delta_{\gamma\delta} + \frac{\lambda+3\mu}{4(\lambda+2\mu)}\,(\delta_{\alpha\gamma}\delta_{\beta\delta}+\delta_{\alpha\delta}\delta_{\beta\gamma}), \tag{9}$$

which yields the following constrained strain within the inclusion

$$\begin{aligned}\epsilon^c_{\alpha\beta} &= \frac{\lambda-\mu}{4(\lambda+2\mu)}\,\delta_{\alpha\beta}\,\epsilon^*_{\gamma\gamma} + \frac{\lambda+3\mu}{2(\lambda+2\mu)}\,\epsilon^*_{\alpha\beta}\\ &= \frac{M\,(\lambda+\mu)}{2\,(\lambda+2\mu)}\,\delta_{\alpha\beta} + \frac{q\,(\lambda+3\mu)}{2\,(\lambda+2\mu)}\,(2\,\hat{n}_\alpha\hat{n}_\beta-\delta_{\alpha\beta}).\end{aligned} \tag{10}$$

Thanks to the linearity of the system, the corresponding velocity, i.e. instantaneous displacement, within the inclusion can now be computed as

$$\begin{aligned}v^{in}_\alpha(\mathbf{x}) = \epsilon^c_{\alpha\beta}x_\beta &= \frac{\lambda-\mu}{4(\lambda+2\mu)}\,\epsilon^*_{\beta\beta}\,x_\alpha + \frac{\lambda+3\mu}{2(\lambda+2\mu)}\,\epsilon^*_{\alpha\beta}\,x_\beta\\ &= \frac{M\,(\lambda+\mu)}{2\,(\lambda+2\mu)}\,x_\alpha + \frac{q\,(\lambda+3\mu)}{2\,(\lambda+2\mu)}\,(2\,\hat{n}_\alpha\,(\hat{n}_\beta x_\beta)-x_\alpha).\end{aligned} \tag{11}$$

The velocity outside of the inclusion will satisfy the biharmonic equation $\nabla^2\nabla^2 v^{out}_\alpha(\mathbf{x}) = 0$, subject to continuity at the surface of the inclusion and must also tend to zero as $r\to\infty$ where $r \equiv ||\mathbf{x}||$. Recalling the radial solutions of the biharmonic equation in 2D (i.e., 1, $\ln r$, r^2, and $r^2\ln r$), we construct the general solution by considering all combinations of derivatives of the radial solutions that are linear in the eigenstrain, tend to zero at infinity, and transform like a vector field:

$$v^{out}_\alpha(\mathbf{x}) = A\,\epsilon^*_{\alpha\beta}\,\frac{\partial\ln r}{\partial x_\beta} + B\,\epsilon^*_{\beta\beta}\,\frac{\partial \ln r}{\partial x_\alpha} + C\,\epsilon^*_{\beta\gamma}\,\frac{\partial^3\ln r}{\partial x_\alpha\partial x_\beta\partial x_\gamma} + D\,\epsilon^*_{\beta\gamma}\,\frac{\partial^3(r^2\ln r)}{\partial x_\alpha\partial x_\beta\partial x_\gamma} \tag{12}$$

where A, B, C, and D are constants to be determined. Recall that the velocity outside of the inclusion is a solution to the equation

$$\mu\,\partial^2_\beta\,v^{out}_\alpha(\mathbf{x}) + (\mu+\lambda)\,\partial_\alpha(\partial_\beta\,v^{out}_\beta(\mathbf{x})) = 0. \tag{13}$$

The fact that $\nabla^2\nabla^2 v^{out}_\alpha(\mathbf{x}) = 0$ is a necessary, but insufficient condition on any solution of Eq. (13). With this fact in mind, we begin to calculate the constants by first computing

$$\frac{\partial^2 v^{out}_\alpha}{\partial x_\beta\partial x_\beta} = D\,\epsilon^*_{\eta\lambda}\,\frac{\partial^3}{\partial x_\alpha\partial x_\eta\partial x_\lambda}\left[\frac{\partial^2(r^2\ln r)}{\partial x_\beta\partial x_\beta}\right] = 4\,D\,\epsilon^*_{\eta\lambda}\frac{\partial^3\ln r}{\partial x_\alpha\partial x_\eta\partial x_\lambda}. \tag{14}$$

In the first equality, we used the identity

$$\frac{\partial^2\ln r}{\partial x_\beta\partial x_\beta} = 0 \tag{15}$$

to cancel the terms proportional to A, B, and C. In the second equality, we made use of the fact that

$$\frac{\partial^2 (r^2 \ln r)}{\partial x_\beta \partial x_\beta} = 4\,(\ln r + 1). \tag{16}$$

Next we compute

$$\begin{aligned}
\frac{\partial^2 v_\beta^{out}}{\partial x_\alpha \partial x_\beta} &= \frac{\partial}{\partial x_\alpha}\Bigg[A\,\epsilon^*_{\beta\eta}\,\frac{\partial^2 \ln r}{\partial x_\beta \partial x_\eta} + B\,\epsilon^*_{\eta\eta}\,\frac{\partial^2 \ln r}{\partial x_\beta \partial x_\beta} + \\
&\qquad C\,\epsilon^*_{\eta\lambda}\,\frac{\partial^4 \ln r}{\partial x_\beta \partial x_\beta \partial x_\eta \partial x_\lambda} + D\,\epsilon^*_{\eta\lambda}\,\frac{\partial^4 (r^2 \ln r)}{\partial x_\beta \partial x_\beta \partial x_\eta \partial x_\lambda}\Bigg] \\
&= \frac{\partial}{\partial x_\alpha}\left[A\,\epsilon^*_{\beta\eta}\,\frac{\partial^2 \ln r}{\partial x_\beta \partial x_\eta} + 4\,D\,\epsilon^*_{\eta\lambda}\,\frac{\partial^2 (\ln r + 1)}{\partial x_\eta \partial x_\lambda}\right] \\
&= (A + 4D)\,\epsilon^*_{\eta\lambda}\,\frac{\partial^3 \ln r}{\partial x_\alpha \partial x_\eta \partial x_\lambda},
\end{aligned} \tag{17}$$

where we have once again made use of both Eq. (15) and Eq. (16). Substituting these results into Eq. (13) yields

$$4\,\mu\,D\,\epsilon^*_{\eta\lambda}\frac{\partial^3 \ln r}{\partial x_\alpha \partial x_\eta \partial x_\lambda} + (\lambda+\mu)\,(A+4D)\,\epsilon^*_{\eta\lambda}\,\frac{\partial^3 \ln r}{\partial x_\alpha \partial x_\eta \partial x_\lambda} = 0, \tag{18}$$

which, when simplified, reveals that

$$D = -\frac{A\,(\lambda+\mu)}{4\,(\lambda+2\mu)}. \tag{19}$$

The velocity outside the inclusion can therefore be written as

$$v_\alpha^{out}(\mathbf{x}) = A\,\epsilon^*_{\alpha\beta}\,\frac{\partial \ln r}{\partial x_\beta} + B\,\epsilon^*_{\beta\beta}\,\frac{\partial \ln r}{\partial x_\alpha} + C\,\epsilon^*_{\beta\gamma}\,\frac{\partial^3 \ln r}{\partial x_\alpha \partial x_\beta \partial x_\gamma} - \frac{A\,(\lambda+\mu)}{4\,(\lambda+2\mu)}\,\epsilon^*_{\beta\gamma}\,\frac{\partial^3 (r^2 \ln r)}{\partial x_\alpha \partial x_\beta \partial x_\gamma}. \tag{20}$$

The following identities are now required to make further progress:

$$\frac{\partial \ln r}{\partial x_\beta} = \frac{x_\beta}{r^2}, \tag{21}$$

$$\frac{\partial^3 \ln r}{\partial x_\alpha \partial x_\beta \partial x_\gamma} = \frac{-2r^2(x_\alpha\delta_{\beta\gamma} + x_\beta\delta_{\alpha\gamma} + x_\gamma\delta_{\alpha\beta}) + 8x_\alpha x_\beta x_\gamma}{r^6}, \tag{22}$$

$$\frac{\partial^3 (r^2 \ln r)}{\partial x_\alpha \partial x_\beta \partial x_\gamma} = \frac{2r^2(x_\alpha\delta_{\beta\gamma} + x_\beta\delta_{\alpha\gamma} + x_\gamma\delta_{\alpha\beta}) - 4x_\alpha x_\beta x_\gamma}{r^4}. \tag{23}$$

Substituting these identities directly into Eq. (20) and simplifying allows us to re-write the velocity in the following form

$$\begin{aligned}
v_\alpha^{out}(\mathbf{x}) &= \left[\frac{A}{r^2} - \frac{4C}{r^4} - \frac{A(\lambda+\mu)}{(\lambda+2\mu)r^2}\right]\epsilon^*_{\alpha\beta}x_\beta + \\
&\qquad \left[\frac{B}{r^2} - \frac{2C}{r^4} - \frac{A(\lambda+\mu)}{2(\lambda+2\mu)r^2}\right]\epsilon^*_{\beta\beta}x_\alpha + \left[\frac{8C}{r^6} + \frac{A(\lambda+\mu)}{(\lambda+2\mu)r^4}\right]\epsilon^*_{\beta\gamma}x_\alpha x_\beta x_\gamma.
\end{aligned} \tag{24}$$

The remaining constants can be computed by enforcing continuity of the velocity field at the boundary of the inclusion where $r = a$. First, we know that the third term that is cubic in $\mathbf{x}$ must vanish when $r = a$ since the velocity within the inclusion is linear in $\mathbf{x}$.

$$\frac{8C}{r^6} + \frac{A(\lambda+\mu)}{(\lambda+2\mu)r^4} = 0 \implies C = -\frac{a^2 A(\lambda+\mu)}{8(\lambda+2\mu)}. \tag{25}$$

The dependence of $\mathbf{v}^{out}(\mathbf{x})$ on C can now be removed yielding

$$\begin{aligned} v_\alpha^{out}(\mathbf{x}) = &\frac{A}{2(\lambda+2\mu)r^2}\left[2\mu + \frac{a^2(\lambda+\mu)}{r^2}\right]\epsilon^*_{\alpha\beta}x_\beta \\ &+ \frac{1}{4r^2(\lambda+2\mu)}\left[-2(A(\lambda+\mu) - 2B(\lambda+2\mu)) + \frac{a^2A(\lambda+\mu)}{r^2}\right]\epsilon^*_{\beta\beta}x_\alpha \\ &+ \frac{A(r^2-a^2)(\lambda+\mu)}{(\lambda+2\mu)r^6}\epsilon^*_{\beta\gamma}x_\alpha x_\beta x_\gamma. \end{aligned} \tag{26}$$

Referencing Eq. (11), we continue to enforce continuity at the boundary of the inclusion and match the coefficients of the velocities inside and outside the inclusion term by term. The term proportional to $\epsilon^* x_\beta$ tells us

$$\frac{A}{2(\lambda+2\mu)a^2}\left(2\mu + (\lambda+\mu)\right) = \frac{\lambda+3\mu}{2(\lambda+2\mu)} \implies A = a^2, \tag{27}$$

and the term proportional to $\epsilon^*_{\beta\beta}x_\alpha$ tells us

$$\begin{aligned} &\frac{1}{4a^2(\lambda+2\mu)}\left[-2(A(\lambda+\mu) - 2B(\lambda+2\mu)) + A(\lambda+\mu)\right] = \\ &\frac{1}{4a^2(\lambda+2\mu)}\left[-2(a^2(\lambda+\mu) - 2B(\lambda+2\mu)) + a^2(\lambda+\mu)\right] = \frac{\lambda-\mu}{4(\lambda+2\mu)} \\ &\implies B = \frac{a^2\lambda}{2(\lambda+2\mu)}. \end{aligned} \tag{28}$$

Finally we arrive at the following form for the velocity outside of the inclusion

$$\begin{aligned} v_\alpha^{out}(\mathbf{x}) = &\frac{q(\lambda+\mu)}{2(\lambda+2\mu)}\left(\frac{a^2}{r^2}\right)\left(\frac{2\mu}{\lambda+\mu} + \frac{a^2}{r^2}\right)(2\hat{n}_\alpha(\hat{n}_\beta x_\beta) - x_\alpha) \\ &+ \frac{M(\lambda+\mu)}{2(\lambda+2\mu)}\left(\frac{a^2}{r^2}\right)^2 x_\alpha \\ &+ \frac{\lambda+\mu}{\lambda+2\mu}\left(\frac{a^2}{r^2}\right)\left(1 - \frac{a^2}{r^2}\right)\left(\frac{M}{2} + q\left(\frac{2(\hat{n}_\beta x_\beta)^2}{r^2} - 1\right)\right)x_\alpha. \end{aligned} \tag{29}$$

In summary, the instantaneous velocity induced by a cell division is has the following

vectorial form

$$\mathbf{v}(\mathbf{x}) = \begin{cases} \frac{M\,(\lambda+\mu)}{2\,(\lambda+2\mu)}\,\mathbf{x} + \frac{q\,(\lambda+3\mu)}{2\,(\lambda+2\mu)}\,(2\,\hat{\mathbf{n}}\,(\hat{\mathbf{n}}\cdot\mathbf{x}) - \mathbf{x}) & \text{for } r \le a \\ \frac{q(\lambda+\mu)}{2(\lambda+2\mu)}\,\left(\frac{a^2}{r^2}\right)\left(\frac{2\mu}{\lambda+\mu} + \frac{a^2}{r^2}\right)(2\,\hat{\mathbf{n}}\,(\hat{\mathbf{n}}\cdot\mathbf{x}) - \mathbf{x}) & \\ +\frac{M(\lambda+\mu)}{2(\lambda+2\mu)}\left(\frac{a^2}{r^2}\right)^2\mathbf{x} & \text{for } r > a \\ +\frac{\lambda+\mu}{\lambda+2\mu}\left(\frac{a^2}{r^2}\right)\left(1-\frac{a^2}{r^2}\right)\left(\frac{M}{2} + q\left(\frac{2\,(\hat{\mathbf{n}}\cdot\mathbf{x})^2}{r^2} - 1\right)\right)\mathbf{x}, & \end{cases} \tag{30}$$

or, in terms of the effective viscosities $\nu_1 = \tau_R\,\mu$ and $\nu_2 = \tau_R\,(\lambda+\mu)$

$$\mathbf{v}(\mathbf{x}) = \begin{cases} \frac{M\,\nu_2}{2\,(\nu_1+\nu_2)}\,\mathbf{x} + \frac{q\,(2\,\nu_1+\nu_2)}{2\,(\nu_1+\nu_2)}\,(2\,\hat{\mathbf{n}}\,(\hat{\mathbf{n}}\cdot\mathbf{x}) - \mathbf{x}) & \text{for } r \le a \\ \frac{q\,\nu_2}{2\,(\nu_1+\nu_2)}\,\left(\frac{a^2}{r^2}\right)\left(\frac{2\,\nu_1}{\nu_2} + \frac{a^2}{r^2}\right)(2\,\hat{\mathbf{n}}\,(\hat{\mathbf{n}}\cdot\mathbf{x}) - \mathbf{x}) & \\ +\frac{M\,\nu_2}{2\,(\nu_1+\nu_2)}\left(\frac{a^2}{r^2}\right)^2\mathbf{x} & \text{for } r > a \\ +\frac{\nu_2}{\nu_1+\nu_2}\left(\frac{a^2}{r^2}\right)\left(1-\frac{a^2}{r^2}\right)\left(\frac{M}{2} + q\left(\frac{2\,(\hat{\mathbf{n}}\cdot\mathbf{x})^2}{r^2} - 1\right)\right)\mathbf{x}. & \end{cases} \tag{31}$$

This equation can be non-dimensionalized by introducing the dimensionless parameter

$$\tilde{\nu} \equiv \frac{\lambda}{2\,(\lambda+\mu)} = \frac{\nu_2 - \nu_1}{2\,\nu_2}, \tag{32}$$

in terms of which the division velocity is

$$\mathbf{v}(\mathbf{x}) = \begin{cases} \frac{M}{4\,(1-\tilde{\nu})}\,\mathbf{x} + \frac{q\,(3-4\,\tilde{\nu})}{4\,(1-\tilde{\nu})}\,(2\,\hat{\mathbf{n}}\,(\hat{\mathbf{n}}\cdot\mathbf{x}) - \mathbf{x}) & \text{for } r \le a \\ \frac{q}{4\,(1-\tilde{\nu})}\,\left(\frac{a^2}{r^2}\right)\left(2\,(1-2\,\tilde{\nu}) + \frac{a^2}{r^2}\right)(2\,\hat{\mathbf{n}}\,(\hat{\mathbf{n}}\cdot\mathbf{x}) - \mathbf{x}) & \\ +\frac{M}{4\,(1-\tilde{\nu})}\left(\frac{a^2}{r^2}\right)^2\mathbf{x} & \text{for } r > a \\ +\frac{1}{2\,(1-\tilde{\nu})}\left(\frac{a^2}{r^2}\right)\left(1-\frac{a^2}{r^2}\right)\left(\frac{M}{2} + q\left(\frac{2\,(\hat{\mathbf{n}}\cdot\mathbf{x})^2}{r^2} - 1\right)\right)\mathbf{x}. & \end{cases} \tag{33}$$

We note that the parameter $\tilde{\nu}$ is not the same as the Poisson ratio introduced in Eq. 11 of the Methods

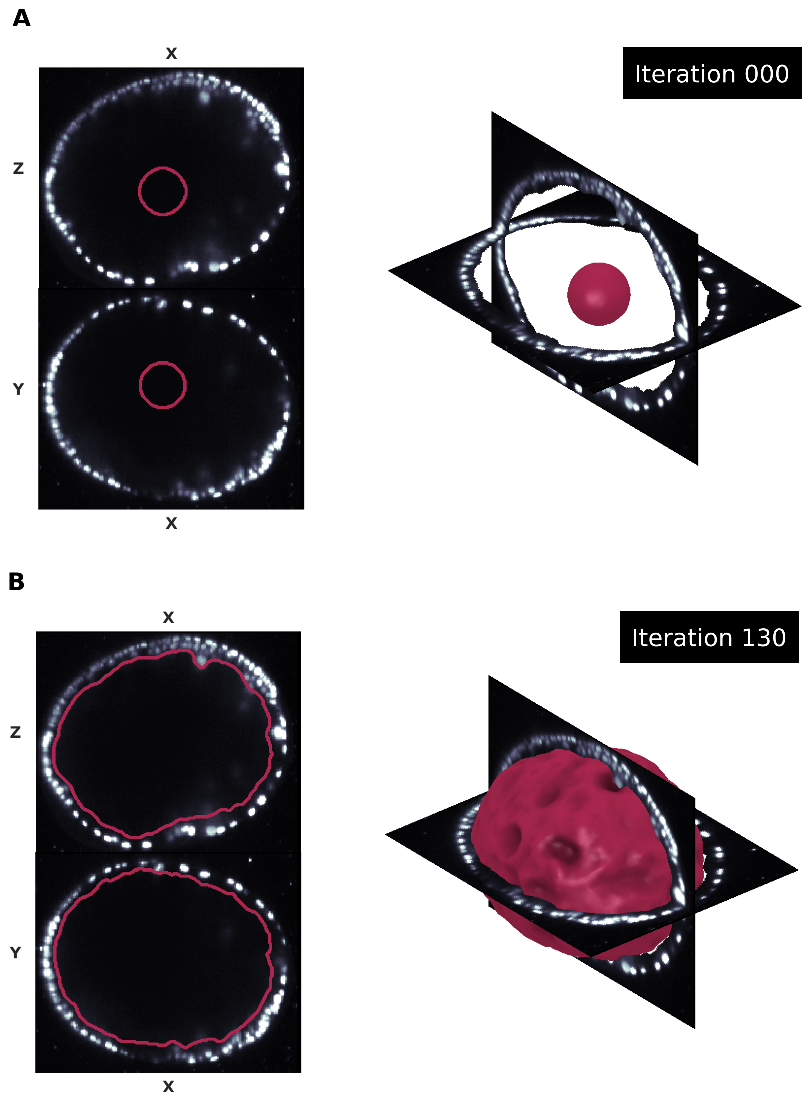

Fig. S1. Surface extraction via level sets. An illustration of the morphological snakes method used to extract the surface of interest in the growing embryo. (**A**) The spherical initial condition (zero completed iterations). (**B**) The segmented surface.

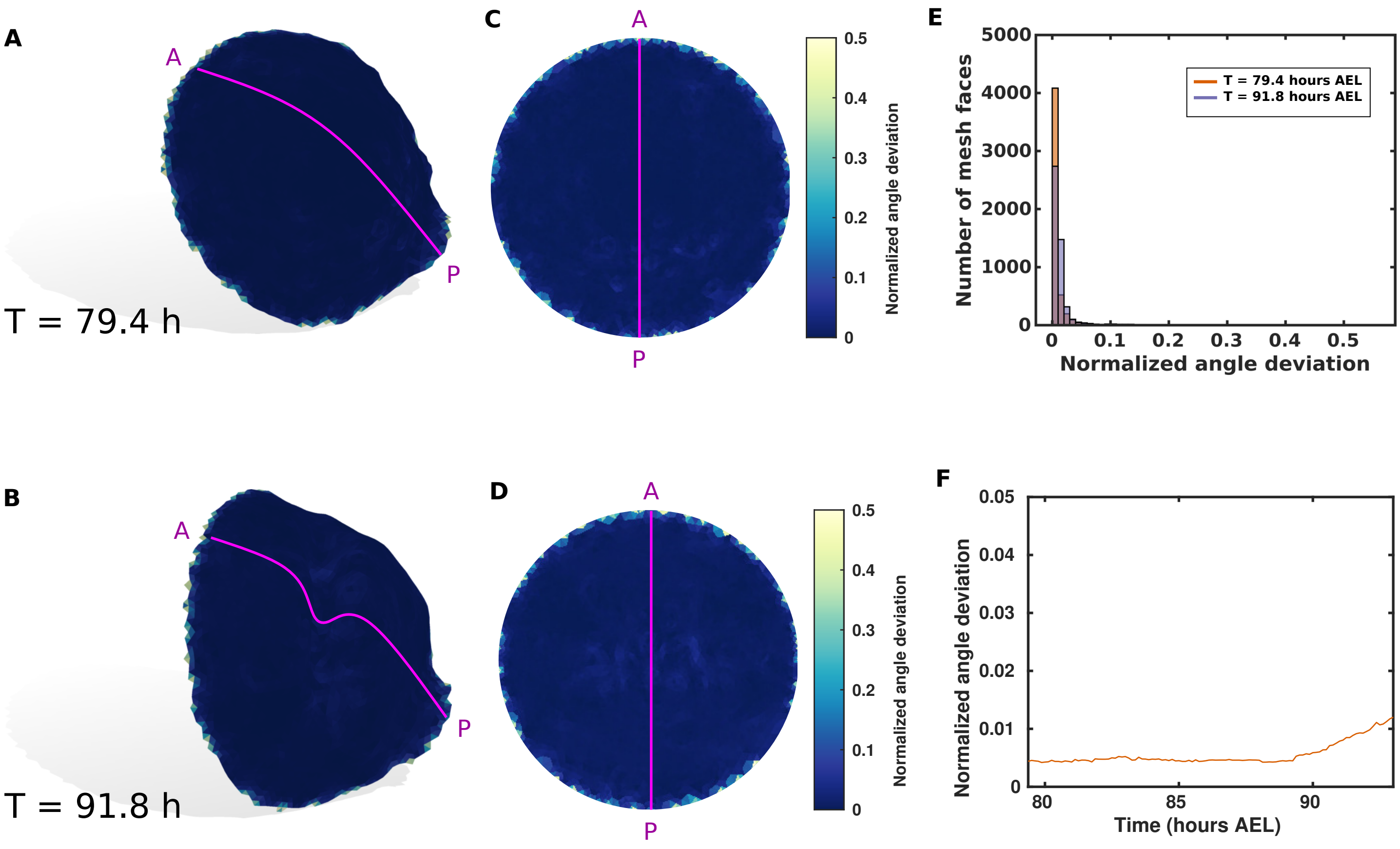

Fig. S2. Discrete conformal parameterization of 3D surfaces. The results of our discrete Ricci flow method in producing conformal parameterizations of the midsurface of the growing ectoderm. Analysis performed on a single embryo. (**A** to **D**) Normalized angle deviation, $(|\theta_1^{3D} - \theta_1^{2D}| + |\theta_2^{3D} - \theta_2^{2D}| + |\theta_3^{3D} - \theta_3^{2D}|)/\pi$, defined on each mesh face reporting the conformality of the mapping. The quantities $\theta_i^{2D}, \theta_i^{3D}$ are the ith internal angle of each face in 2D and 3D, respectively. Mapping remains almost perfectly conformal in the bulk of the mesh, even at late times when the curvature of the tail fold is pronounced. (A) Normalized angle deviation on the 3D surface at T = 79.4 hours AEL. (B) Normalized angle deviation on the 3D surface at 91.9 hours AEL. (C) Normalized angle deviation displayed in the 2D pullback space at T = 79.4 hours AEL. (D) Normalized angle deviation displayed in the 2D pullback space at 91.8 hours AEL. (**E**) Histograms of the normalized angle deviation at the two representative time points. (**F**) Median normalized angle deviation over the full time course of the data set.

A B

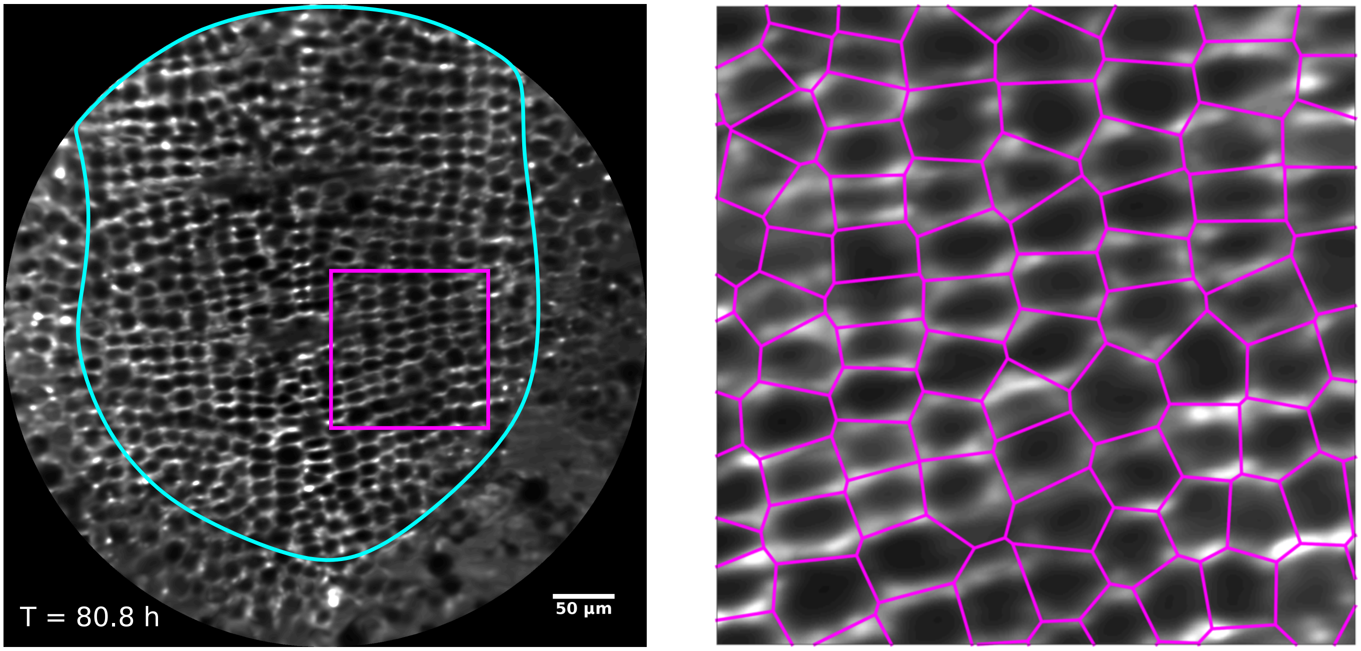

Fig. S3. Visualizing cell membranes in the growing embryo. (**A**) The trunk segmental ectoderm pulled to the plane and visualized with a lipid dye. Outer cyan marking delineates embryo from extra embryonic tissue. Inner magenta marking delineates the boundary of the region visualized in Fig. S3B. (**B**) A close up visualization of a region of the trunk segmental ectoderm and a comparison to the Voronoi tessellation produced using the cell centers extracted from our cell segmentation pipelines.

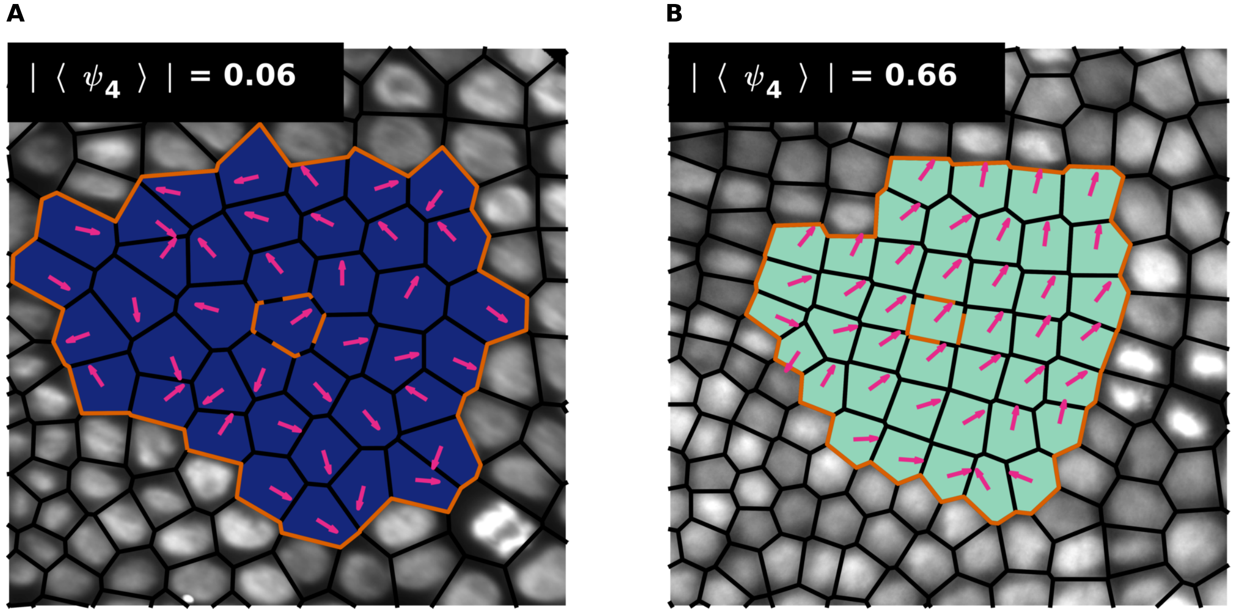

Fig. S4. Effects of averaging the discrete fourfold orientational order parameter. (**A** and **B**) Average fourfold order parameter over the third order natural neighborhood of the single cells shown in (Fig. 2, B and C). The dotted orange boundary denotes the seed cell. The solid orange boundary denotes the averaging region. Arrows indicate the orientation of the single cell order parameter for each cell in the averaging region. (A) Average order parameter in a disordered region. (B) Average order parameter in a highly fourfold ordered region. In general, small differences in orientation will result in significantly lower mean order parameters compared to the magnitude of the single cell order parameters. This effect is especially noticeable in the high order case.

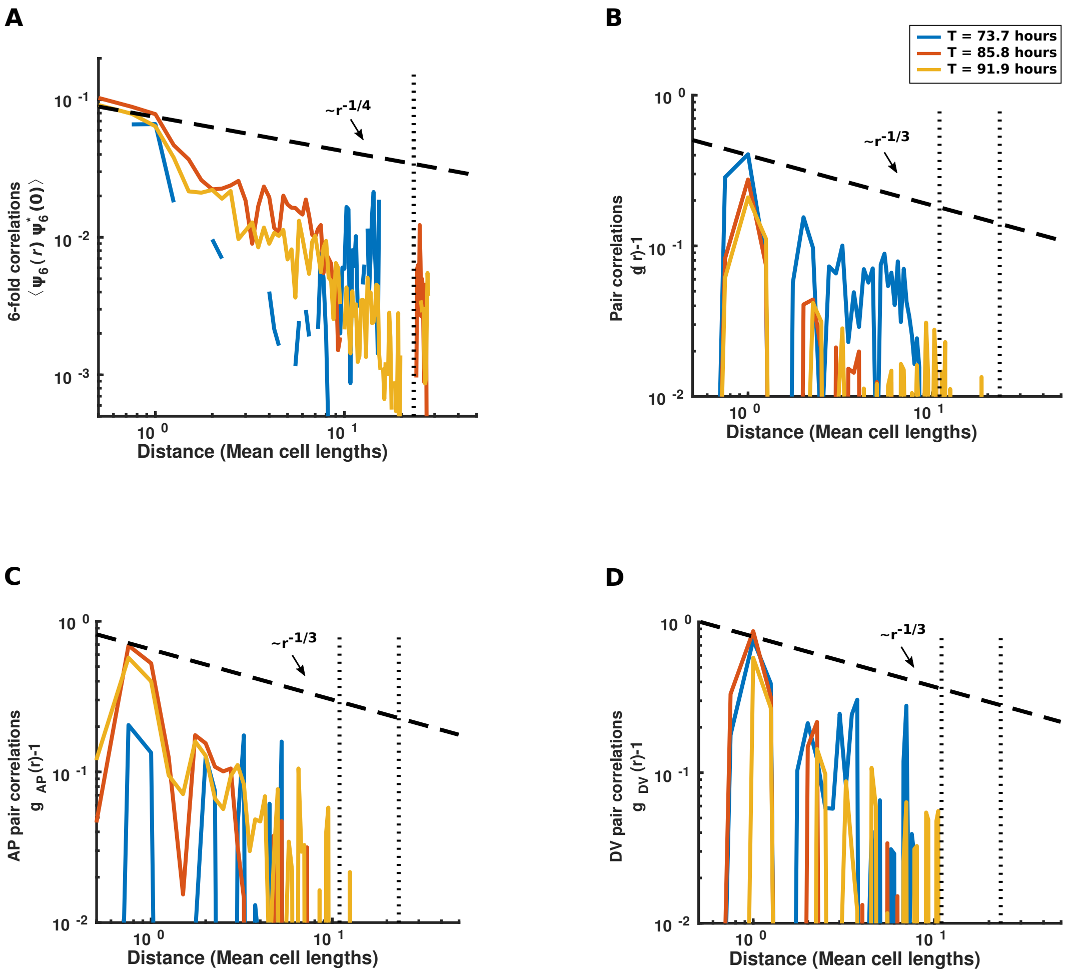

Fig. S5. Trunk ectodermal germband exhibits neither sixfold nor translational/smectic order. Measured values extracted from a single embryo. (**A**) The two-point sixfold orientational correlation functions at three representative time points. All time points exhibit exponential decay. Vertical dotted line indicates largest lateral length scale in the system. (**B**) The isotropic pair correlation function. Vertical dotted lines indicate length scale of the system along the A-P and D-V axes. All time points exhibit exponential decay, implying no isotropic translational order. (**C -D**) Anisotropic variations of the pair correlation function. All time points for both variations decay exponentially. The lack of anisotropic translational order along any preferred direction preclude the possibility of smectic order in the system. (C) The pair correlation function measured along the D-V axis. (D) The pair correlation function measured along the A-P axis.

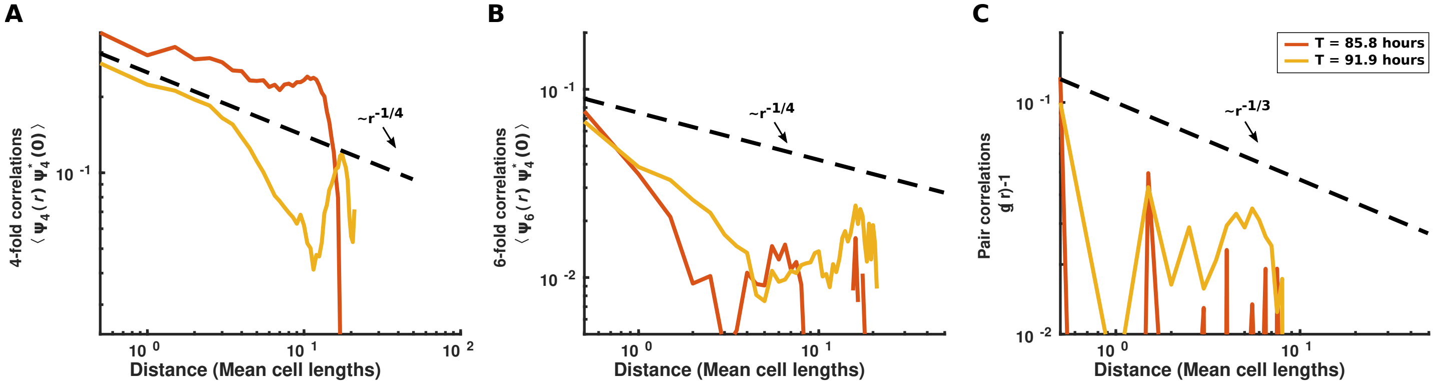

Fig. S6. Trunk ectodermal germband exhibits neither sixfold nor translational/smectic order (cont.). Orientational order correlation functions and isotropic pair correlation function for a separate embryo at the same representative times. (**A**) The two-point fourfold orientational correlation functions at two representative time points. Tissue exhibits quasi-long range order at the at T = 85.8 h. (**B**) The two-point sixfold orientational correlation functions at three representative time points. All time points exhibit exponential decay. (**C**) The isotropic pair correlation function. All time points exhibit exponential decay, implying no isotropic translational order.

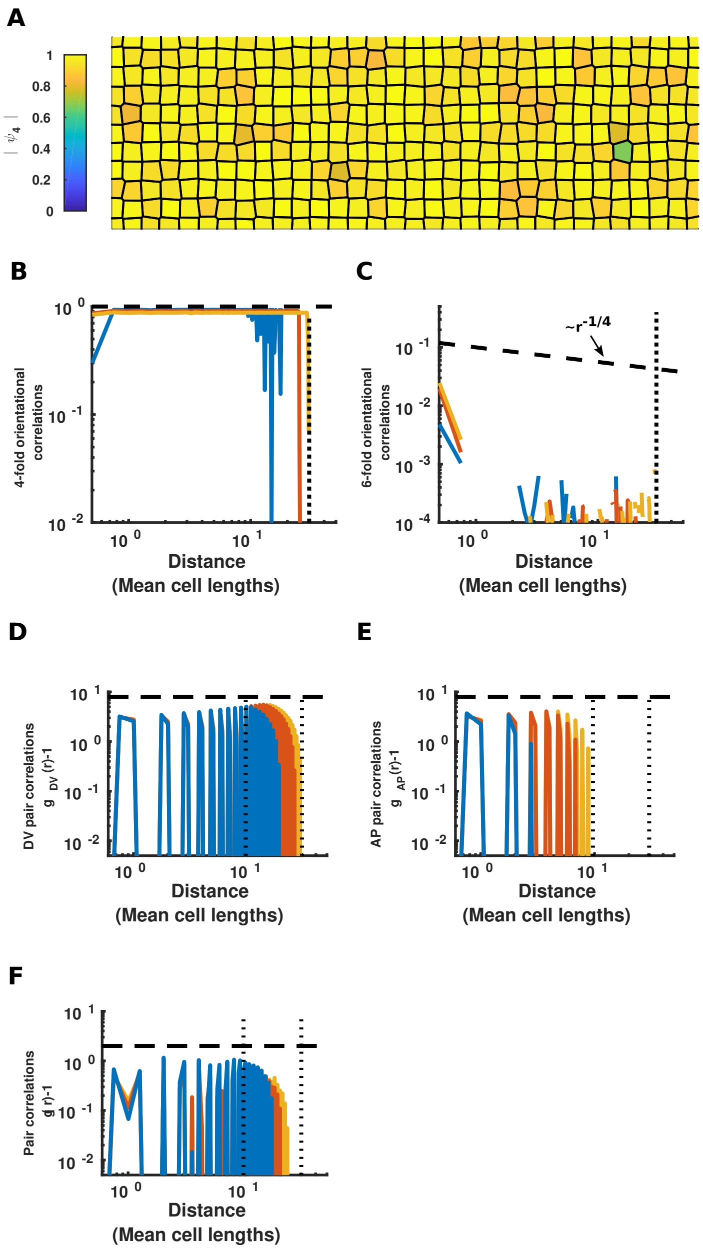

Fig. S7. The germband does not exhibit translational order. (**A**) An example translationally ordered synthetic data set with the same spatial dimensions and cell density as the germband at $T = 91.9$ hours after egg lay. Color of cells indicates the magnitude of the fourfold orientational order parameters. (**B** to **F**) Correlation functions for synthetic data sets with the same system sizes and cell densities as the representative time points in Fig. 2G and Fig. S5. (B) The two-point fourfold orientational correlation function. (C) The two-point sixfold orientational correlation function. (D) The pairwise radial distribution function measured along the D-V axis of the synthetic data set. The horizontal dashed line is a guide for the eye. (E) The pairwise radial distribution function measured along the A-P axis of the synthetic data set. (F) Radially symmetric pair correlation function

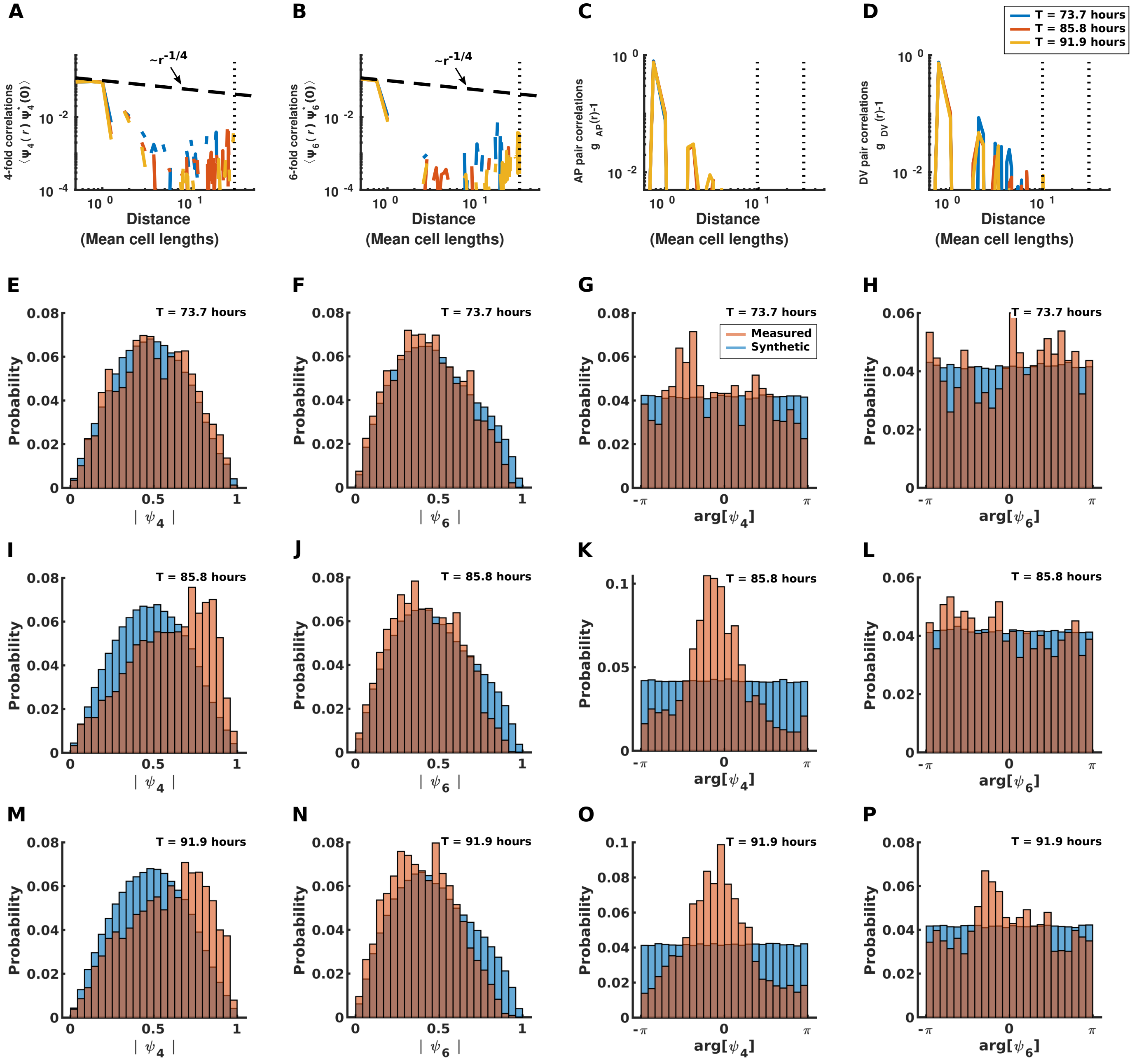

Fig. S8. Observed order in the embryo is significant compared to disordered synthetic data sets. (**A** to **D**) Correlation functions for synthetic data sets with the same system sizes and cell densities as the representative time points in Fig. 2G and Fig. S5. Curves report the mean correlation function values for $N = 1000$ independent synthetic data sets generated according to the parameters of each representative time point. (A) The two-point fourfold orientational correlation function. (B) The two-point sixfold orientational correlation function. (C) The pairwise radial distribution function measured along the A-P axis of the synthetic data set. (D) The pairwise radial distribution function measured along the D-V axis of the synthetic data set. (**E** to **H**) Comparison of the fourfold and sixfold order parameters of the measured and synthetic data sets at early times. Histogram

of synthetic values pooled from $N = 1000$ synthetic data sets. (E) The magnitude of the fourfold order parameter. (F) The magnitude of the sixfold order parameter. (G) The phase of the fourfold order parameter. (H) The phase of the sixfold order parameter. (**I** to **L**) Comparison of the fourfold and sixfold order parameters of the measured and synthetic data sets at intermediate times. Histogram of synthetic values pooled from $N = 1000$ synthetic data sets. (I) The magnitude of the fourfold order parameter. (J) The magnitude of the sixfold order parameter. (K) The phase of the fourfold order parameter. (L) The phase of the sixfold order parameter. (**M** to **P**) Comparison of the fourfold and sixfold order parameters of the measured and synthetic data sets at late times. Histogram of synthetic values pooled from $N = 1000$ synthetic data sets. (M) The magnitude of the fourfold order parameter. (N) The magnitude of the sixfold order parameter. (O) The phase of the fourfold order parameter. (P) The phase of the sixfold order parameter.

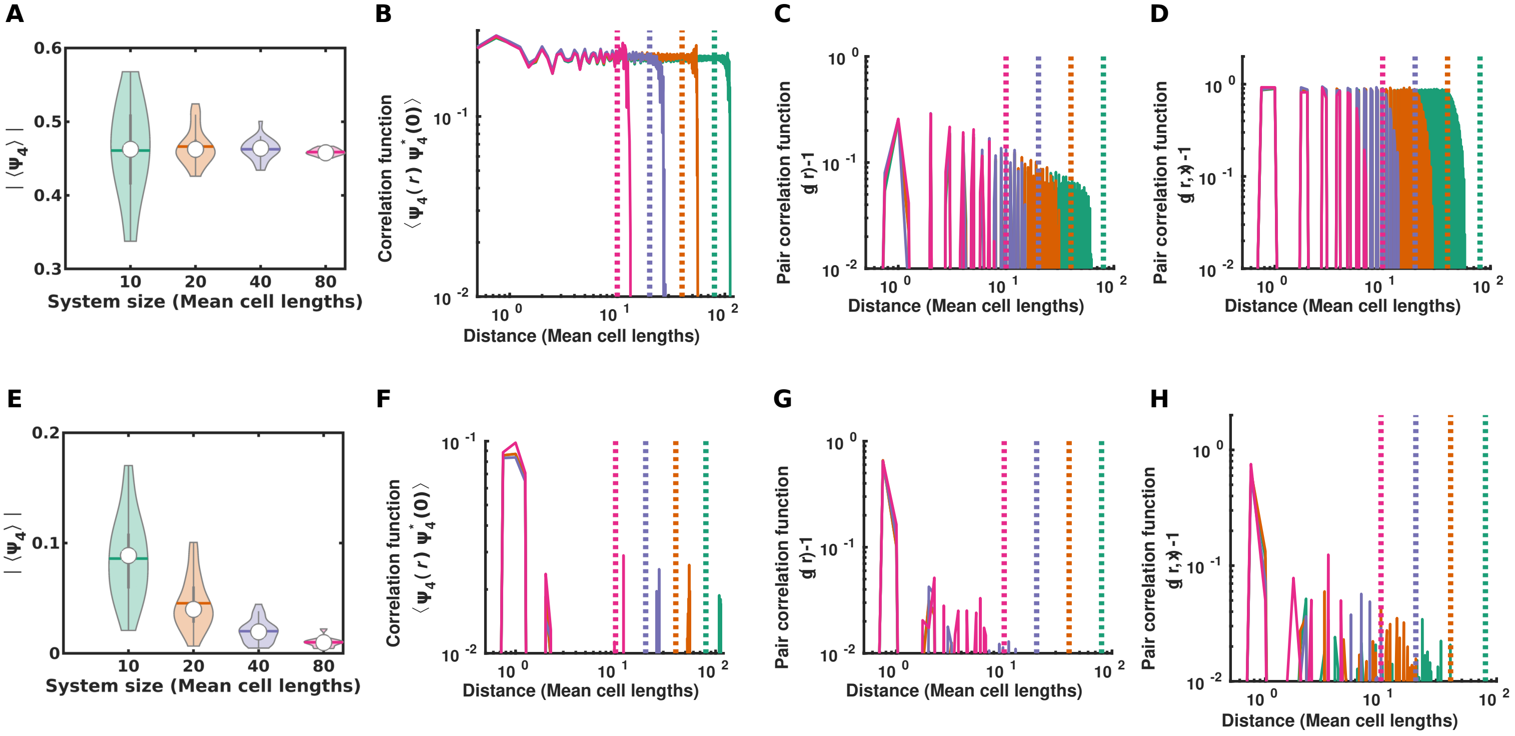

Fig. S9. Finite size effects on orientational and translational order. Finite size effects were investigated using synthetic data sets describing both fourfold ordered (**A**-**D**) and totally disordered systems (**E**-**H**). Systems were generated with square aspect ratios and system sizes $L = 10, 20, 40, 80$ cell lengths. $N = 25$ synthetic data sets for each system size. (**A**) Distributions of the mean global fourfold order parameter $|\langle\psi_4\rangle|$ for an orientationally ordered system. Distributions are much more closely packed around the mean for larger systems, but the mean of the distributions are constant for all system sizes. Circle markers denote distribution medians and bold horizontal lines denote distribution means. (**B**) Fourfold orientational correlation function for an ordered system. Vertical dotted lines indicate system size. Correlations approach and maintain a constant value over the entirety of the system. (**C**) Radially symmetric pair correlation function $g(r) - 1$. Pair correlation function decays algebraically over most of the system size. (**D**) Horizontal cut of the pair correlation function $g(r, x) - 1$. Correlations also decay algebraically, albeit extremely slowly. (**E**) Distributions of the mean global fourfold order parameter $|\langle\psi_4\rangle|$ for a disordered system. The mean value for each distribution is more variable across system size than in the ordered case, but all values are still consistent with a disordered system. Mean values appear to monotonically approach zero as system size in increased. Circle markers denote distribution medians and bold horizontal lines denote distribution means. (**F**) Fourfold orientational correlation functions decay exponentially for all system sizes. Some erroneous spikes are observed at distances larger than the system size. (**G**) Radially symmetric pair correlation function $g(r) - 1$ decays exponentially for all system sizes. (**H**) Horizontal cut of the pair correlation function $g(r, x) - 1$ decays exponentially for all system sizes.

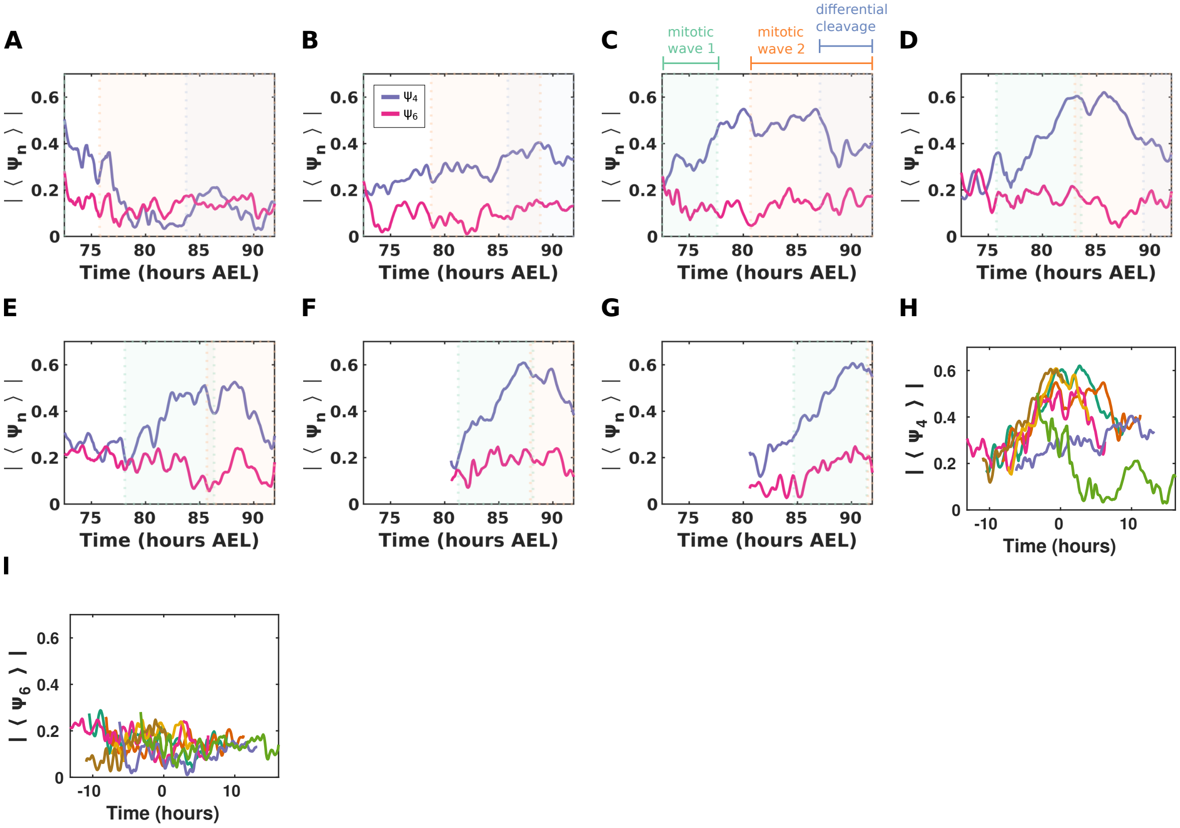

Fig. S10. Temporal profile of orientational order is similar across different parasegments. All parasegments belong to the same single embryo. (**A** to **G**) Per-parasegment profile of of the magnitude of the average fourfold and sixfold orientational order parameters over time. Ordering of panels (A to G) mirrors anterior to posterior ordering of the physical segments (i.e. the parasegment shown in (A) is anterior to the parasegment shown in (B), etc.). (**H**) The magnitude of the average fourfold orientational order parameter for each parasegment shifted in time so that $T = 0$ corresponds to the first cell division of mitotic wave 2. (**I**) The magnitude of the average sixfold orientational order parameter for each parasegment shifted in time so that $T = 0$ corresponds to the first cell division of mitotic wave 2.

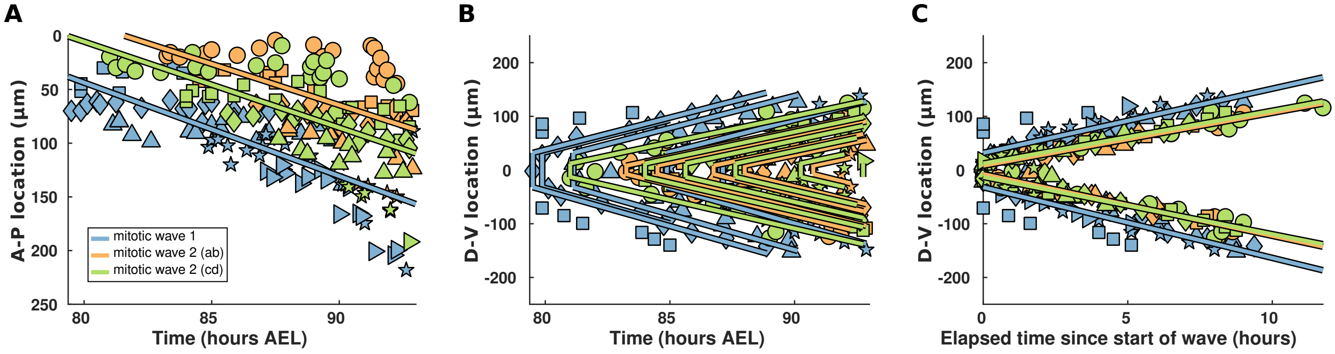

Fig. S11. Division wave pattern is preserved across embryos. (**A** to **C**) The location of mitotic wave division events over time in a different embryo than the one shown in (Fig 3, D to F). Shapes indicate the parasegment within which a division occurs. Indicated lines are linear fits to all division events associated to a particular mitotic wave. (A) shows the location of each division along the A-P axis. The speed of mitotic wave 1 is 8.7 ± 0.8 μm/hr. The speed of mitotic wave 2 (AB) is 7.7 ± 1.6 μm/hr. The speed of mitotic wave 2 (CD) is 8.0 ± 1.2 μm/hr. (B) and (C) show the location of each division along the D-V axis. Division times in (C) have been normalized to the occurrence of the first division event associated with a particular wave in a specific parasegment. The speed of mitotic wave 1 is 12.6 ± 1.1 μm/hr. The speed of mitotic wave 2 (AB) is 10.7 ± 0.5 μm/hr. The speed of mitotic wave 2 (CD) is 10.3 ± 0.6 μm/hr.

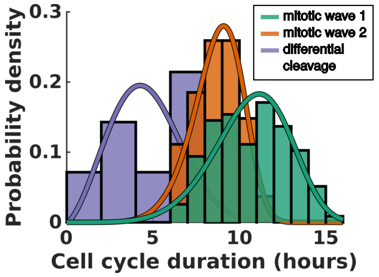

Fig. S12. Distribution of cell cycle durations throughout growth. Solid lines are Weibull distributions fit to the cell cycle duration histograms. Average duration and standard deviation for mitotic wave 1 is 10.7 ± 2.2 hours from $N = 117$ cell cycles. Average duration and standard deviation for mitotic wave 2 is 8.7 ± 1.5 hours from $N = 27$ cell cycles. Average duration and standard deviation for differential cleavage is 4.6 ± 2.0 hours from $N = 7$ cell cycles. All cell cycles drawn from the same single embryo.

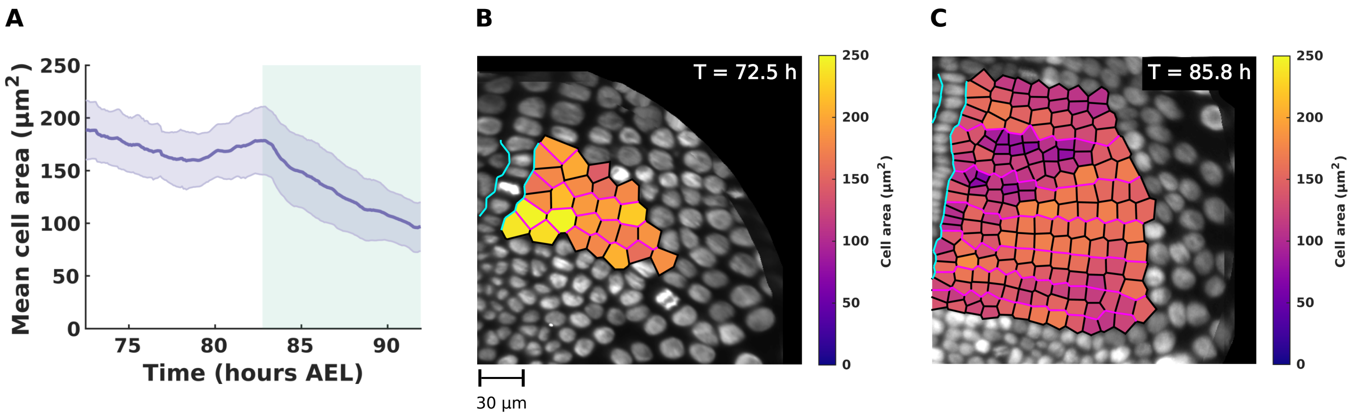

Fig. S13. True cell areas are accurately captured using tissue cartography (**A**) The mean cell area over time. Shaded region shows standard deviation. Cell areas drawn from a single embryo. (**B** and **C**) Cell areas at two representative time points in the left ectodermal compartment. Cell size varies primarily as a function of timing in the cell cycle (recently divided cells are smaller - see Fig. S21B). However, cell size is essentially uniform in space at each time point for cells in similar points in their respective cell cycles.

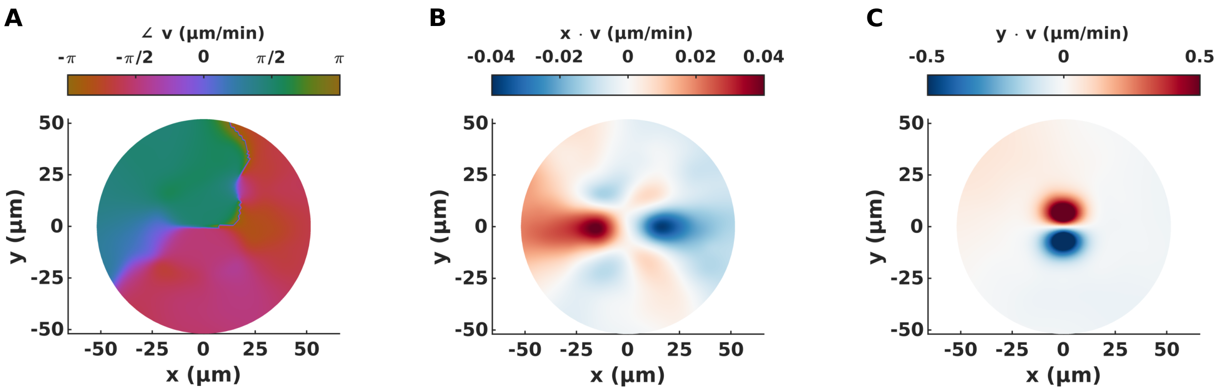

Fig. S14. The velocity of the mean cell division event. Analysis performed on 190 division events drawn from a single embryo. (**A**) The orientation of the velocity of the mean cell division event. (**B**) The x-component of the velocity of the mean cell division event. (**C**) The y-component of the velocity of the mean cell division event.

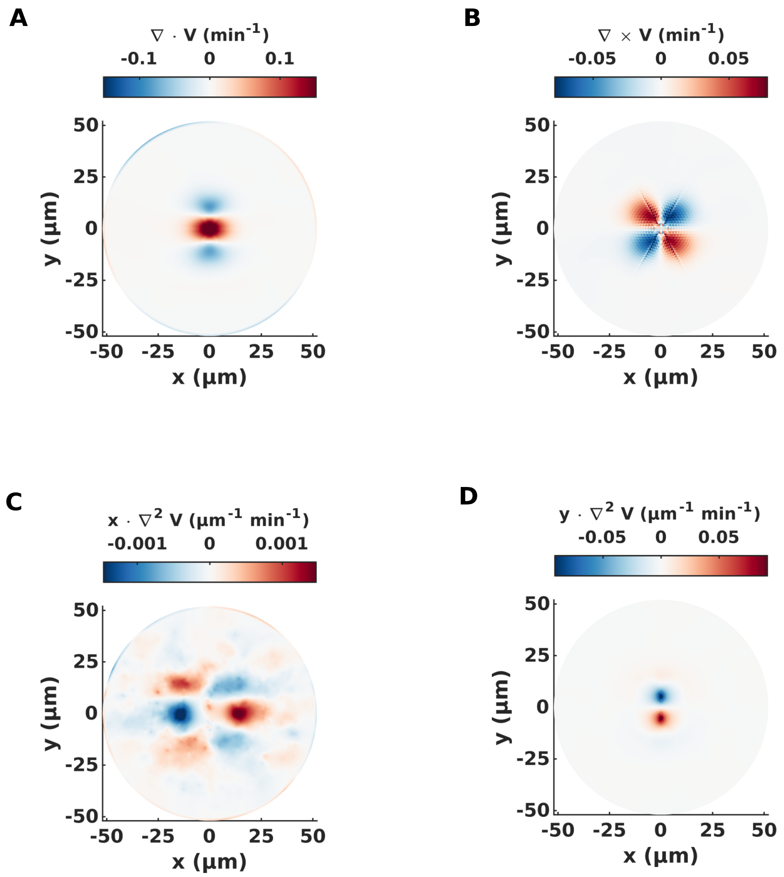

Fig. S15. Gradients of the velocity of the mean cell division event. Analysis performed on 190 division events drawn from a single embryo. (**A**) The divergence of the mean cell division velocity. (**B**) The 'curl' of the mean cell division velocity. (**C**) The x-component of the Laplacian of the mean cell division velocity. (**D**) The y-component of the Laplacian of the mean cell division velocity.

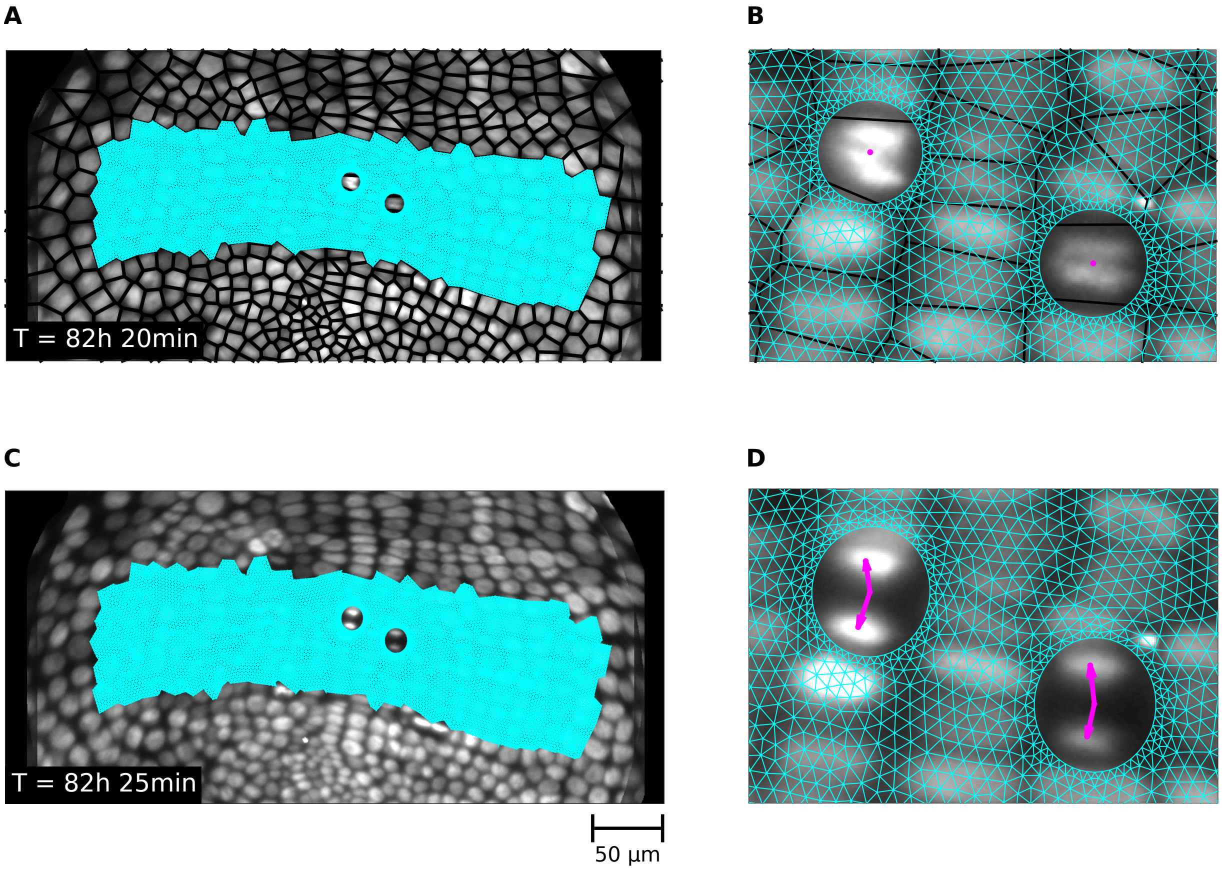

Fig. S16. Numerical solution of the fluid mechanical equations of motion. Examples of the triangular mesh used to implement a finite element method (FEM) solution to the equations of motion predicting tissue velocities from cell divisions. (**A**) The undeformed mesh triangulation. Circular holes have been removed surrounding cells about to divide. (**B**) A zoomed-in visualization of the undeformed mesh near the cells about to divide. (**C**) The deformed mesh at the subsequent time. Mesh vertex velocities have been scaled by a constant pre-factor (x5) to improve visibility. (**D**) A zoomed-in visualization of the mesh near the recently divided cells. Note that the initially circular holes are deformed into ellipses.

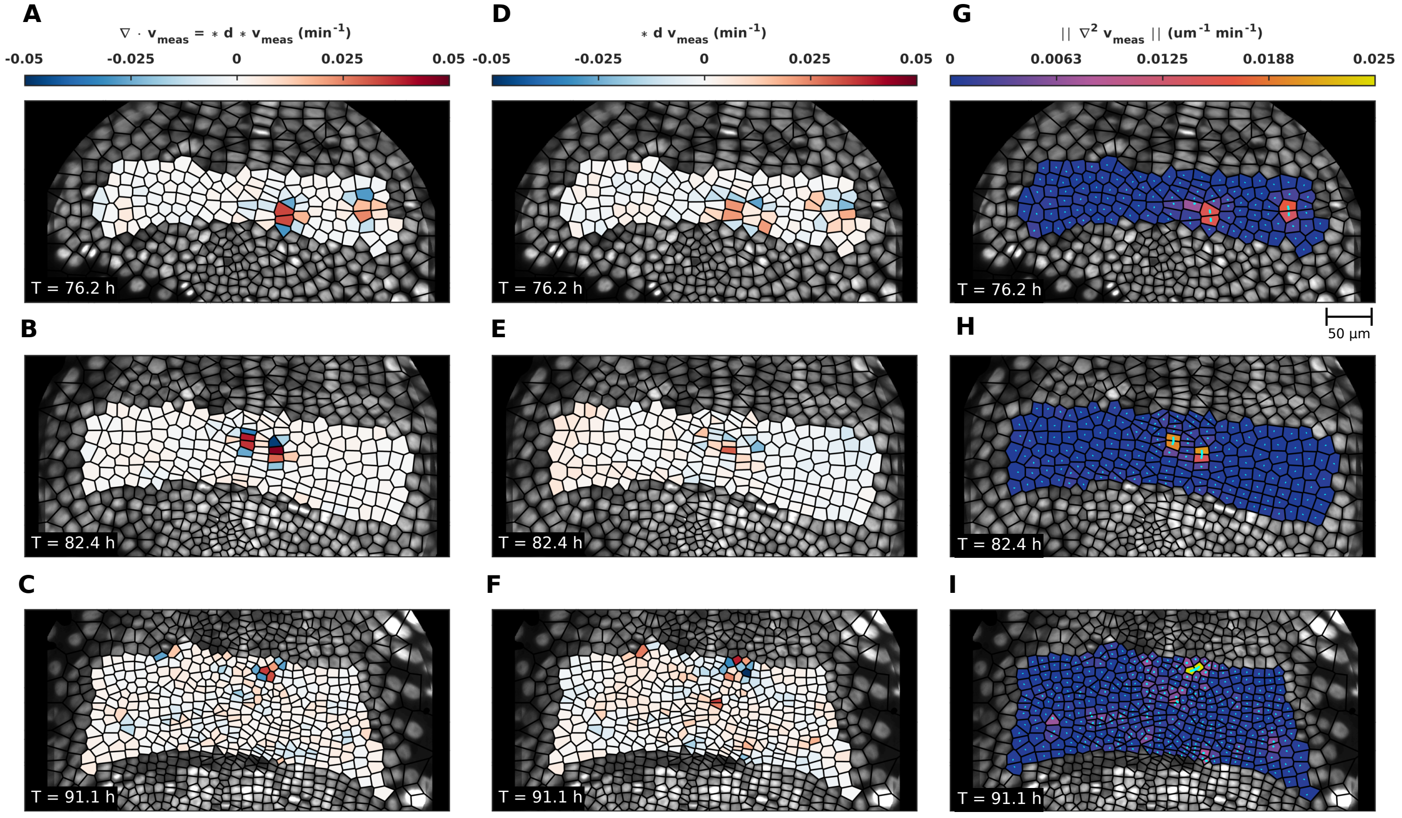

Fig. S17. Gradients of the measured tissue velocity fields. (**A** to **C**) The divergence of the measured tissue velocity fields for three representative time points. Explicit construction in term of exterior derivatives and Hodge stars is shown in (A). (**D** to **F**) The 'curl' of the measured tissue velocity fields for three representative time point. Explicit construction in terms of exterior derivatives and Hodge stars is shown in (D). (**G** to **I**) The Laplacian of the measured tissue velocity fields for three representative time points.

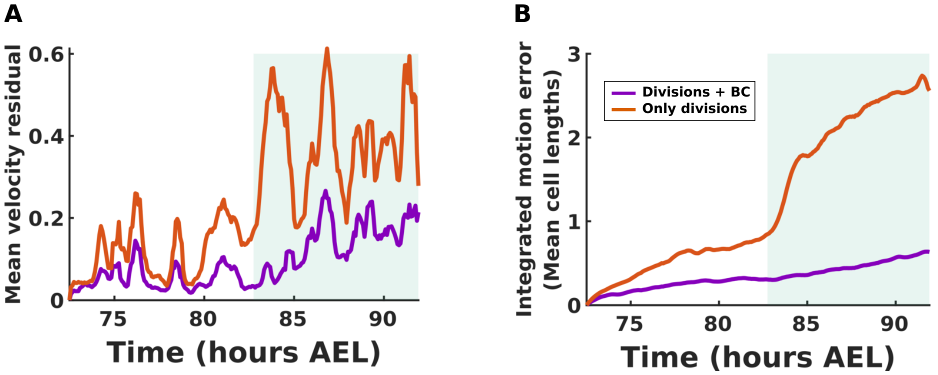

Fig. S18. Hydrodynamic model of cell divisions predicts cell positions during mitotic wave. Analysis performed on a single embryo. (**A**) Mean residual between the measured cell velocities and the cell velocities predicted by the active hydrodynamic model using the measured boundary conditions (purple) and without any imposed boundary conditions (orange), i.e. simply the sum of the velocities predicted using our inclusion model. The prediction with no boundary conditions performs reasonably well during the first stage of growth, but breaks down during convergent extension. (**B**) The error in the predictions for the locations of cell centers generated by integrating measured velocities and calculated velocities, i.e. $||\vec{\mathbf{x}}_{meas}(t) - \vec{\mathbf{x}}_{calc}(t)||$ in units of mean cell lengths. The prediction with no boundary conditions stays within a single cell length of the true position for the first stage of growth, but grows large during convergent extension.

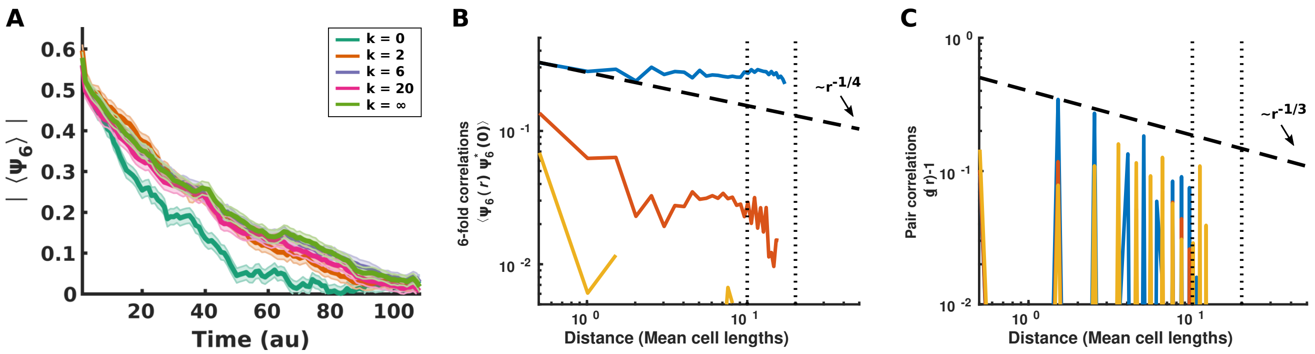

Fig. S19. Additional division wave simulation properties. (**A**) The absolute value of the mean sixfold order parameter during division wave simulations. Different curves correspond to different concentrations k of distributions from which division orientations were randomly drawn (see Fig. 5F). Sixfold order degrades completely for all values of k. Reported values were averaged over the same 5 independent simulations for each value of k shown in Fig. 5D. Shaded regions show standard error. (**B**) The two-point correlation function of the sixfold orientational order parameter generated by division wave simulations with $k = \infty$. Vertical lines indicate the length and width of the tissue, respectively. Initial configurations display quasi-long range sixfold order. (**C**) The isotropic pair correlation function generated by division wave simulations with $k = \infty$. No configuration displays algebraically decaying order over the entire tissue indicating that the initial and final configurations are sixfold and fourfold orientationally ordered, respectively, but not translationally ordered.

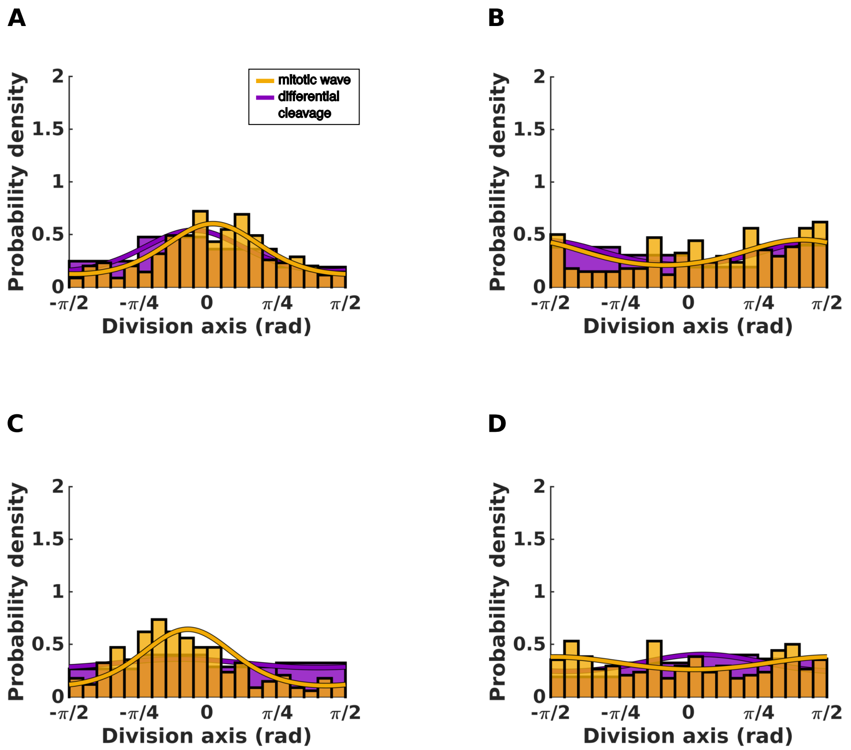

Fig. S20. Orientations of cell divisions are not predicted by local mechanical or geometric signals. Analysis performed on 222 mitotic wave divisions and 67 differential cleavage divisions drawn from a single embryo. (**A**) Histogram of orientations of cell divisions relative to the principal axis of the strain-rate of the corresponding parent cell integrated over the hour preceding cell division. (**B**) Histogram of the orientations of cell divisions relative to the axis of elongation of the corresponding parent cell averaged over the hour preceding cell division. (**C**) Histogram of the orientations of cell divisions relative of the orientation of the fourfold order parameter of the corresponding parent cell averaged over the hour preceding cell division. (**D**) Histogram of the orientations of cell divisions relative to the orientation of the sixfold order parameter of the corresponding parent cell averaged over the hour preceding cell division.

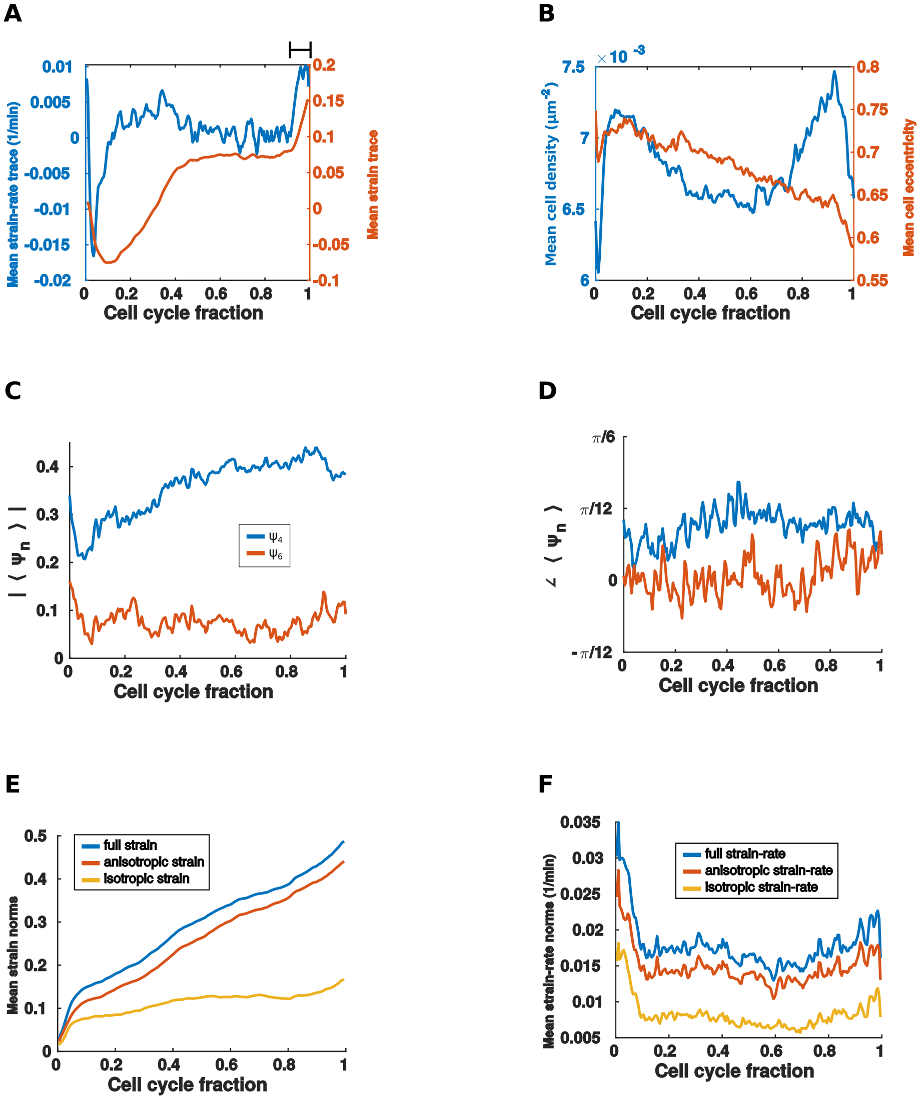

Fig. S21. Timing of cell divisions are not predicted by local mechanical or geometric signals. Analysis performed on 184 cell cycles drawn from a single embryo. (**A** to **F**) Various geometric and mechanical fields averaged over a normalized cell cycle. Average includes cell cycles from both mitotic waves and differential cleavage. (A) The trace of the mean strain-rate tensor (left) and the trace of the mean cumulative strain tensor (right). The black bar in the upper right hand corner indicates the growth phase preceding cell division, i.e. cell division has already been initiated in this phase. (B) Mean cell density (left) and mean eccentricity of an ellipse fit to each cell (right). (C) The magnitude of the average fourfold and sixfold order parameters. (D) The orientation of the average fourfold and sixfold order parameters relative to the A-P axis. (E) The Frobenius norm of the average full cumulative strain tensor, the anistropic part of the the average strain tensor, and the isotropic part of the average strain tensor. (F) The Frobenius norm of the full average strain-rate tensor, the anisotropic part of the average strain-rate tensor, and the isotropic part of the average strain-rate tensor.

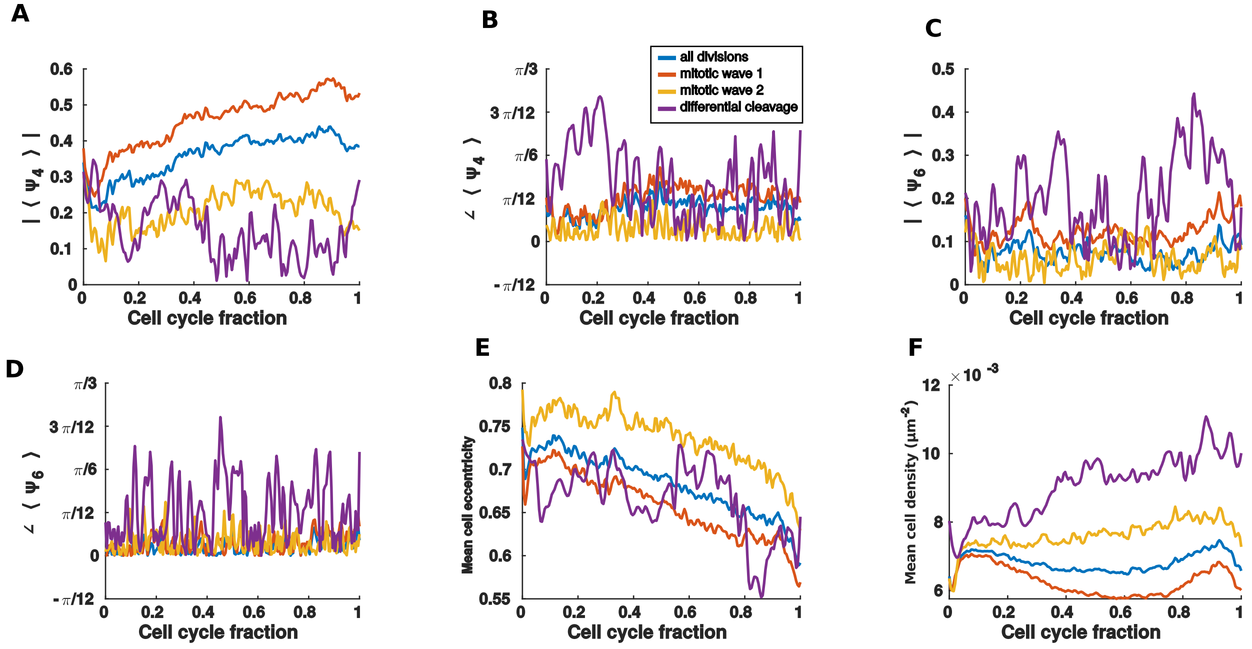

Fig. S22. Timing of cell divisions are not predicted by local geometric signals (cont.). Analysis performed on 184 cell cycles drawn from a single embryo. (**A** to **F**) Various geometric fields averaged over a normalized cell cycle. Separate averages are included for each mitotic wave and differential cleavage. (A) The magnitude of the mean fourfold orientational order parmeter. (B) The orientation of the mean fourfold orientational order parmeter. (C) The magnitude of the mean sixfold orientational order parameter. (D) The orietnation of the mean sixfold orientational order parameter. (E) The mean eccentricity of an ellipse fit to each cell. (F) The mean cell density.

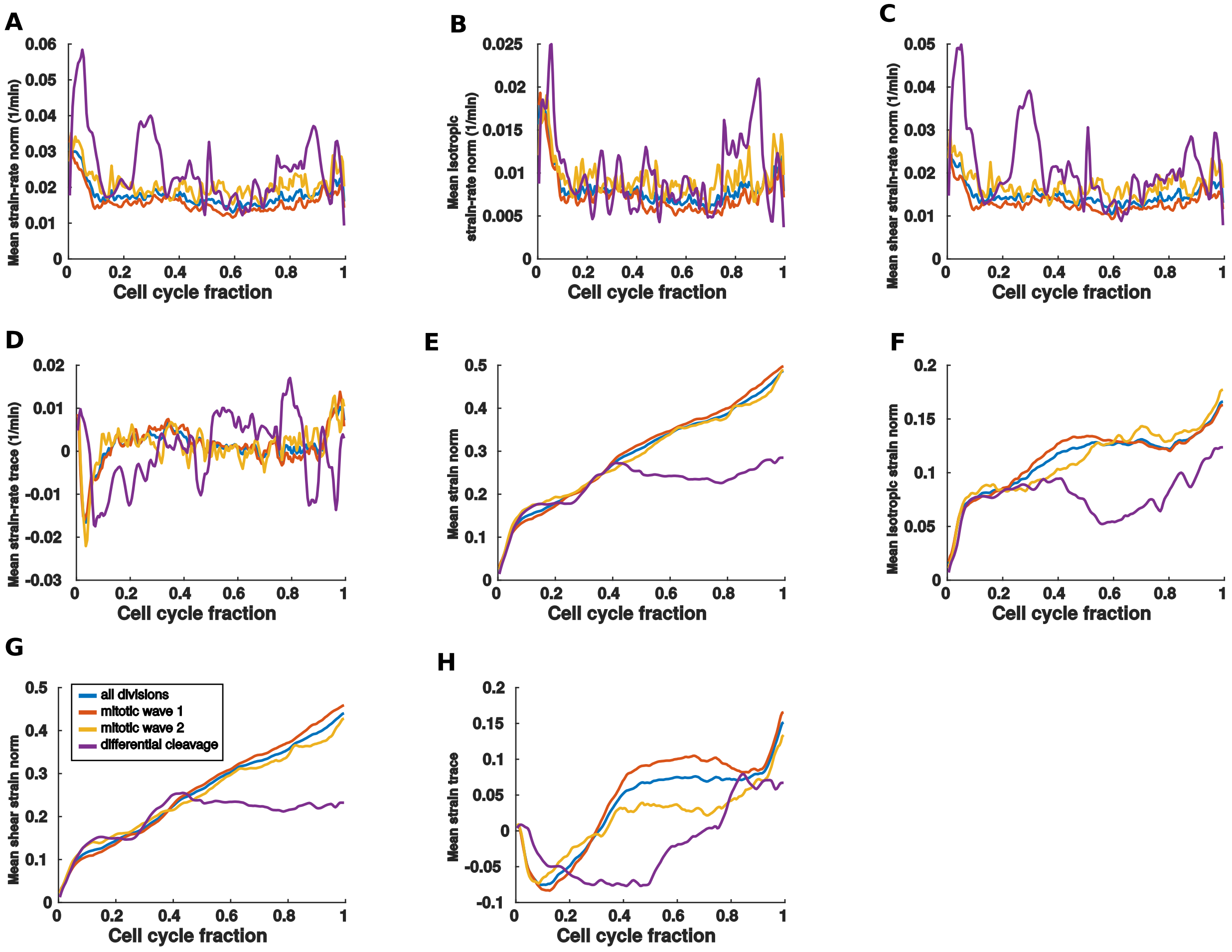

Fig. S23. Timing of cell divisions are not predicted by local mechanical signals (cont.). Analysis performed on 184 cell cycles drawn from a single embryo. (**A** to **H**) Various mechanical fields averaged over a normalized cell cycle. Separate averages are included for each mitotic wave and differential cleavage. (A) The Frobenius norm of the mean strain-rate tensor. (B) The Frobenius norm of the isotropic part of the mean strain-rate tensor. (C) The Frobenius of the anisotropic part of the mean strain-rate tensor. (D) The trace of the mean strain-rate tensor. (E) The Frobenius norm of the mean cumulative strain tensor. (F) The Frobenius norm of the isotropic part of the mean cumulative strain tensor. (G) The Frobenius norm of the anisotropic part of the mean cumulative strain tensor. (H) The trace of the mean cumulative strain tensor.

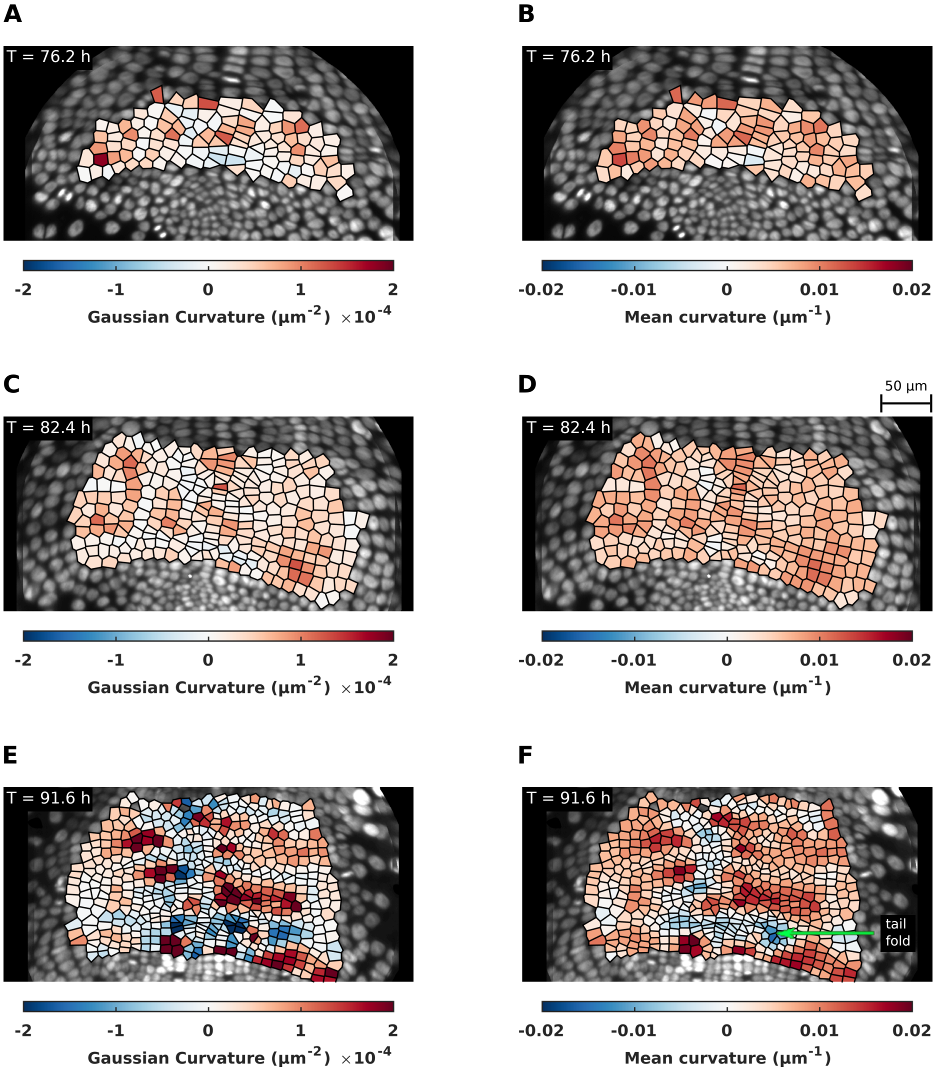

Fig. S24. Surface curvatures in the growing *Parhyale* embryo. (**A**) Per-cell Gaussian curvature at early times. (**B**) Per-cell mean curvature at early times. (**C**) Per-cell Gaussian curvature at intermediate times. (**D**) Per-cell mean curvature at intermediate times. (**E**) Per-cell Gaussian curvature at late times. (**F**) Per-cell mean curvature at late times.

	p-Value	K-S Statistic
$T = 73.7\,\mathrm{h}:\ \lvert\psi_4\rvert$	9.23e-05	0.0475
$T = 73.7\,\mathrm{h}:\ \arg[\psi_4]$	5.64e-08	0.0627
$T = 73.7\,\mathrm{h}:\ \lvert\psi_6\rvert$	1.11e-06	0.0571
$T = 73.7\,\mathrm{h}:\ \arg[\psi_6]$	2.50e-06	0.0555
$T = 85.8\,\mathrm{h}:\ \lvert\psi_4\rvert$	1.05e-106	0.2016
$T = 85.8\,\mathrm{h}:\ \arg[\psi_4]$	7.55e-100	0.1949
$T = 85.8\,\mathrm{h}:\ \lvert\psi_6\rvert$	6.61e-20	0.0863
$T = 85.8\,\mathrm{h}:\ \arg[\psi_6]$	2.21e-06	0.0477
$T = 91.9\,\mathrm{h}:\ \lvert\psi_4\rvert$	2.62e-95	0.1804
$T = 91.9\,\mathrm{h}:\ \arg[\psi_4]$	4.86e-89	0.1744
$T = 91.9\,\mathrm{h}:\ \lvert\psi_6\rvert$	3.45e-41	0.1183
$T = 91.9\,\mathrm{h}:\ \arg[\psi_6]$	1.12e-07	0.0499

Table S1. Statistical comparison of orientational order in measured and synthetic data sets. The Kolmogorov-Smirnov (K-S) statistic and asymptotic p-values for the distributions shown in Fig. S8. Details on calculation in methods.

References

1. Pan, Y., Heemskerk, I., Ibar, C., Shraiman, B. I. & Irvine, K. D. Differential growth triggers mechanical feedback that elevates Hippo signaling. *Proceedings of the National Academy of Sciences of the United States of America* **113,** E6974–E6983 (2016).

2. Landau, L., Lifshitz, E., Kosevich, A., Sykes, J., Pitaevskii, L. & Reid, W. *Theory of Elasticity: Volume 7* (Elsevier Science, 1986).

3. Etournay, R., Popović, M., Merkel, M., Nandi, A., Blasse, C., Aigouy, B., Brandl, H., Myers, G., Salbreux, G., Jülicher, F. & Eaton, S. Interplay of cell dynamics and epithelial tension during morphogenesis of the Drosophila pupal wing. *eLife* **4,** e07090 (2015).

4. Streichan, S. J., Lefebvre, M. F., Noll, N., Wieschaus, E. F. & Shraiman, B. I. Global morphogenetic flow is accurately predicted by the spatial distribution of myosin motors. *eLife* **7,** e27454 (2018).

5. Saadaoui, M., Rocancourt, D., Roussel, J., Corson, F. & Gros, J. A tensile ring drives tissue flows to shape the gastrulating amniote embryo. *Science* **367,** 453–458 (2020).

6. Lecuit, T. & Lenne, P.-F. Cell surface mechanics and the control of cell shape, tissue patterns and morphogenesis. *Nature Reviews Molecular Cell Biology* **8,** 633–644 (2007).

7. Wang, N., Tolić-Nørrelykke, I. M., Chen, J., Mijailovich, S. M., Butler, J. P., Fredberg, J. J. & Stamenović, D. Cell prestress. I. Stiffness and prestress are closely associated in adherent contractile cells. *https://doi.org/10.1152/ajpcell.00269.2001* **282,** 606–616 (2002).

8. MacKintosh, F. C. & Levine, A. J. Nonequilibrium Mechanics and Dynamics of Motor-Activated Gels. *Physical Review Letters* **100,** 018104 (2008).

9. Hartsock, A. & Nelson, W. J. Adherens and tight junctions: Structure, function and connections to the actin cytoskeleton. *Biochimica et Biophysica Acta (BBA) - Biomembranes* **1778,** 660–669 (2008).

10. Doostmohammadi, A., Thampi, S. P., Saw, T. B., Lim, C. T., Ladoux, B. & Yeomans, J. M. Celebrating Soft Matter's 10th Anniversary: Cell division: a source of active stress in cellular monolayers. *Soft Matter* **11,** 7328–7336 (2015).

11. Kasza, K. E., Rowat, A. C., Liu, J., Angelini, T. E., Brangwynne, C. P., Koenderink, G. H. & Weitz, D. A. The cell as a material. *Current Opinion in Cell Biology* **19,** 101–107 (2007).

12. Wozniak, M. A. & Chen, C. S. Mechanotransduction in development: a growing role for contractility. *Nature Reviews Molecular Cell Biology* **10,** 34–43 (2009).

13. Farhadifar, R., Röper, J.-C., Aigouy, B., Eaton, S. & Jülicher, F. The Influence of Cell Mechanics, Cell-Cell Interactions, and Proliferation on Epithelial Packing. *Current Biology* **17,** 2095–2104 (2007).

14. Serwane, F., Mongera, A., Rowghanian, P., Kealhofer, D. A., Lucio, A. A., Hockenbery, Z. M. & Campàs, O. In vivo quantification of spatially varying mechanical properties in developing tissues. *Nature Methods* **14,** 181–186 (2017).

15. Noll, N., Mani, M., Heemskerk, I., Streichan, S. J. & Shraiman, B. I. Active tension network model suggests an exotic mechanical state realized in epithelial tissues. *Nature Physics* **13,** 1221–1226 (2017).

16. Brugués, J. & Needleman, D. Physical basis of spindle self-organization. *Proceedings of the National Academy of Sciences* **111,** 18496–18500 (2014).

17. Eshelby, J. D. The determination of the elastic field of an ellipsoidal inclusion, and related problems. *Proceedings of the Royal Society of London. Series A. Mathematical and Physical Sciences* **241,** 376–396 (1957).

18. Sarkar, S., Čebron, M., Brojan, M. & Košmrlj, A. Elastic multipole method for describing deformation of infinite two-dimensional solids with circular inclusions. *Physical Review E* **103,** 053003 (2021).

19. Dasgupta, R., Hentschel, H. G. E. & Procaccia, I. Microscopic mechanism of shear bands in amorphous solids. *Physical Review Letters* **109** (2012).

20. Bar-Sinai, Y., Librandi, G., Bertoldi, K. & Moshe, M. Geometric charges and nonlinear elasticity of two-dimensional elastic metamaterials. *Proceedings of the National Academy of Sciences of the United States of America* **117,** 10195–10202 (2020).
